# Exploring Spin-Phonon Coupling in Magnetic 2D Metal-Organic Frameworks

**DOI:** 10.3390/nano13071172

**Published:** 2023-03-25

**Authors:** Diego López-Alcalá, Alberto M. Ruiz, José J. Baldoví

**Affiliations:** Instituto de Ciencia Molecular, Universitat de València, 46980 Paterna, Spain

**Keywords:** metal-organic frameworks, 2D magnetism, spin-phonon coupling, first principles, coordination chemistry

## Abstract

Layered magnetic metal-organic frameworks (MOFs) are an emerging class of materials that can combine the advantages of both MOFs and 2D magnetic crystals. The recent discovery of large coercivity and long-range magnetic ordering up to 515 K in a layered MOF of general formula MCl_2_(pyz)_2_ (M = transition metal, pyz = pyrazine) offers an exciting versatile platform to achieve high-T_C_ magnetism at the 2D limit. In this work, we investigate the exfoliation feasibility down to the monolayer of VCl_2_(pyz)_2_ and CrCl_2_(pyz)_2_ by means of first-principles calculations. We explore their structural, electronic, magnetic and vibrational properties, as well as the effect of halide substitution. Then, we provide a full analysis of the spin-phonon coupling (SPC) in both 2D derivatives. Our calculations reveal a low SPC and thermal evolution of the magnetic exchange interactions and single-ion anisotropy mainly governed by low-frequency phonon modes. Finally, we provide chemical insights to improve the performance of these magnetic 2D MOFs based on the effective manipulation of the phonon modes that can present a major impact on their magnetic properties.

## 1. Introduction

Metal-Organic Frameworks (MOFs) are emerging porous materials constructed by the interconnection of metal ions and organic linkers. This structural arrangement gives rise to highly versatile coordination compounds with a large variety of astonishing features such as structural, electronic and magnetic tunability, sizable porosity and the presence of very large surfaces [1,2,3]. Thanks to these features, MOFs have applications in a diverse set of fields, for example, gas separation [4], catalysis [5], drug delivery [6] and energy storage [7,8], among others. Importantly, the chemical nature of these crystalline frameworks results in their design *à la carte*, thus leading to the effective tailoring of the desired properties [9]. In addition, post-synthetic modification of their crystallographic coordinates by means of chemical reactivity provides an appealing route for a detailed fine-tuning of their electronic functionalities [10,11].

A particular class that is gaining increasing interest is that of the so-called MOF magnets [12,13]. Compared to classical magnetic materials, these systems present the advantages of MOFs, which allows the regulation of their structural, electronic, or magnetic properties via chemical modifications. This opens new avenues for their application as building blocks for emergent technologies such as spintronics [14] or magnonics [15]. Specifically, MOF magnets with open-shell organic linkers have been shown to display strong magnetic coupling between magnetic moments arising from the *d* orbitals of metal centers and radicals. This situation leads to ultra-high operative temperatures, e.g., T_C_ = 400 K (V(TCNE)_x_ where x ≈ 2) [16] or, more recently, T_C_ = 515 K in a reduced chromium derivative of a family of coordination solids of general formula MCl_2_(pyz)_2_ (M = transition metal; pyz = pyrazine) [17,18,19]. This last example represents the highest ordering temperature exhibited for any bulk octahedrally coordinated metal ion, combined with large magnetic coercivity, and opens a wide range of possibilities to achieve higher critical temperatures.

Moreover, this family of MOF magnets is composed of electrically neutral magnetic layers that are exclusively stacked by van der Waals interactions. This enables the possibility of mechanical exfoliation to a few layers [20,21,22,23], allowing the combination in an atomically-thin material the advantages of both MOFs and 2D magnetic crystals [24,25]. Furthermore, the fast development of on-surface synthetic approaches creates a promising route to achieve a scalable fabrication process [26], whereas long-range magnetic order and electronic communication between the spin carriers in the system can be favored by chemical substitution [27,28]. The survival of long-range magnetic ordering in a limited-layer magnetic material is strongly dependent on the intrinsic spin-phonon coupling (SPC), which is responsible for the magnetic fluctuations and magnon dissipation [29]. However, the investigation of the effect of spin and lattice degrees of freedom in MOF magnets is still in its infancy and deserves to be tackled [30].

Here, we present a first-principles study based on density functional theory (DFT) of the Cr and V derivatives of the family of MCl_2_(pyz)_2_ neutral 2D layered MOFs. First, we determine the feasibility of the exfoliation of these nanomaterials, as well as the effects on the electronic structure when reducing the dimensionality of the system. Then, we substitute the Cl ligands for heavier halides, exploring a promising way to tune their electronic, structural and magnetic properties by means of chemical design. Finally, we systematically investigate the lattice vibrations and corresponding spin-phonon coupling and thermal evolution of magnetic exchange and single-ion anisotropy parameters. This allows us to provide chemical insights for their further rational design to make them operative at higher temperatures.

## 2. Methods

We performed first-principles spin-polarized density functional theory (DFT) calculations as implemented in the QuantumEspresso package [31]. The exchange-correlation energy was described using the generalized gradient approximation (GGA) and the Perdew–Burke–Ernzerhof (PBE) functional [32]. We selected standard ultra-soft solid-state pseudopotentials (USPP) from the QuantumEspresso database. The electronic wave functions were expanded with well-converged kinetic energy cut-offs for the wave functions (charge density) of 80(800) and 90(900) for V and Cr derivatives, respectively. We fully optimized both atomic positions and lattice parameters until the forces on each atom were smaller than 10^−3^ Ry and the energy difference between two consecutive relaxation steps was less than 10^−4^ Ry. To avoid unphysical interactions between images along the non-periodic directions, we added a vacuum of 18 Å in the *z* direction in all single-layer calculations. The Brillouin zone was sampled by a fine Γ-centered 10 × 10 × 1 (10 × 10 × 10) and 20 × 20 × 1 (20 × 20 × 20) k-point Monkhorst–Pack mesh [33] to simulate the monolayer (bulk) of the studied V and Cr-based materials, respectively. In the bulk calculations, we considered van der Waals interactions by adding dispersion corrections by means of the semi-empirical Grimme-D2 approach [34]. To properly describe the electronic structure of all the systems, we adopted a Hubbard-corrected (DFT + U) approach, where U is the on-site Coulomb repulsion, to properly describe the strong correlation of *d* electrons of V and Cr using the simplified version proposed by Dudarev et al. [35].

In addition, we carried out broken-symmetry and vibrational DFT calculations using the Gaussian09 package in its revision D01 [36]. For magnetic exchange calculations, we employed the TPSSh hybrid functional [37] and Def2TZVP basis set [38] in combination with the corresponding density fitting approximation. We computed J values from the total energy difference of the high-spin (HS) and low-spin (LS) cases, as established by Yamaguchi et al. [39]. The vibrational analysis was performed using the PBE0 hybrid-exchange correlation functional [40] in combination with a 6-311G basis set [41,42]. The single-ion anisotropy (D) was obtained at the NEVPT2 level [43] using the Orca package [44,45]. We used the BP86 functional [46] in all calculations. The Def2TZVP basis set [38] was used for V, Cr, and N atoms, while the Def2SVP basis set [38] was used for C and H atoms. In all calculations, we included the corresponding auxiliary basis set.

## 3. Results

VCl_2_(pyz)_2_ and CrCl_2_(pyz)_2_ are layered materials stacked by van der Waals interactions and crystallize in tetragonal *I4/mmm* and orthorhombic *Immm* space groups, respectively. Within each layer, VCl_2_(pyz)_2_ presents a square lattice of pyrazine-bridged metal centers with pyz rings disordered on two positions (*cis-* and *trans*-oriented) and chloride ligands aligned along the *c* crystallographic direction. Meanwhile, CrCl_2_(pyz)_2_ is almost isostructural with VCl_2_(pyz)_2_ but the Cr-Cr and Cr-N bond distances slightly deviate from a perfect 2D square network, as determined from synchrotron X-ray powder diffraction. For CrCl_2_(pyz)_2_, the magnetic intralayer interactions take place between the Cr(III) ions (S = 3/2) and pyrazine radical spins (S = 1/2), both coupled antiferromagnetically, which results in a net ferrimagnetic state (S = 1 per molecule) below ~55 K. In the case of VCl_2_(pyz)_2_, adjacent V(II) metallic centers are coupled antiferromagnetically—mediated by neutral pyrazine ligands—and preserve the global antiferromagnetic behavior up to ~120 K.

**Figure 1:**
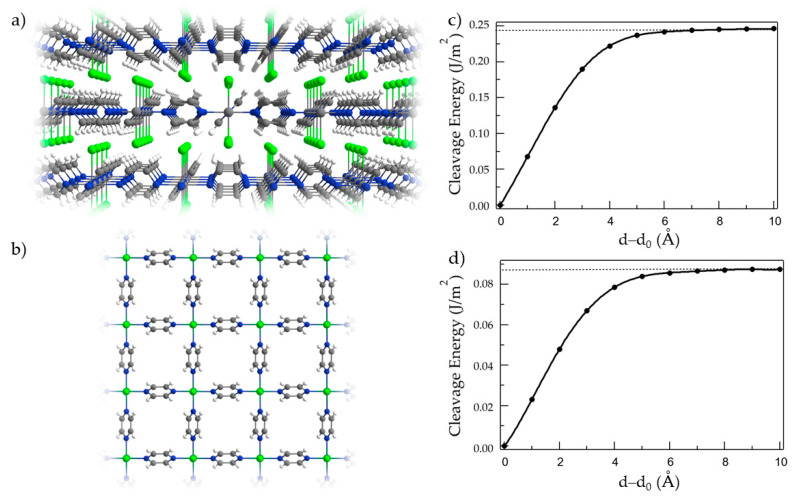
(**a**) Lateral and (**b**) top view of bulk MCl_2_(pyz)_2_. Color code: M (dark green), Cl (green), C (gray), N (blue) and H (white). (**c**,**d**) Cleavage energy as a function of interlayer separation from equilibrium (d_0_) between two (**c**) VCl_2_(pyz)_2_ and (**d**) CrCl_2_(pyz)_2_ monolayers.

Recently, it has been proposed that these layered materials could be easily exfoliated down to the 2D limit due to the neutral nature of the layers [19]. In order to rationalize this possibility and provide insights for their experimental study, we perform systematic total energy calculations as a function of the interlayer distance (d) in a slab formed by two layers, where d_0_ is the optimized separation between layers in the bulk. This allows us to obtain the cleavage energy of both nanomaterials (Figure 1c,d). The cleavage energy is equivalent to the exfoliation energy and thus is defined as the necessary energy to separate a single layer from the bulk material. It directly reflects the strength of the interlayer interactions. As we can observe, the cleavage energy saturates at d-d_0_ ~7 Å, with values of ~0.25 and 0.1 J/m^2^ for VCl_2_(pyz)_2_ and CrCl_2_(pyz), respectively. This indicates that at higher interlayer distances both layers are not interacting anymore and thus the total energy of the system is the result of two independent monolayers. The obtained values are smaller than the calculated for prototypical 2D layered materials such as graphite (0.39 J/m^2^) or MoS_2_ (0.27 J/m^2^) [47] whereas they are similar to other magnetic 2D materials such as CrSBr (0.18 J/m^2^) [48] or CrI_3_ (0.3 J/m^2^) [49]. Therefore, our calculations reveal that both VCl_2_(pyz)_2_ and CrCl_2_(pyz) are likely to be easily exfoliated in the laboratory.

Next, we perform a full optimization of their atomic coordinates and lattice vectors by means of DFT and investigate the effect of halogen substitution in the monolayer. This is done by replacing the Cl ions with Br, thus forming VBr_2_(pyz)_2_ and CrBr_2_(pyz)_2_, and with I, which leads to VI_2_(pyz)_2_ and CrI_2_(pyz)_2_ (see Table S1). Compared with the bulk, the optimized chemical structures of single-layer VCl_2_(pyz)_2_ and CrCl_2_(pyz)_2_ reveal torsion angles of ~25–26°, which implies that the pyrazine rings are less tilted than in their bulk counterparts (~33–35°). This is because van der Waals forces play a major role in the interlayer interaction of the pyrazine rings and stabilize a more planar configuration with respect to the *c*-axis. The analysis of the 2D systems, when moving from Cl to Br and I, evidence an increase in the torsion angles of the pyrazine and the metal-halogen distances due to stronger repulsion interactions between the atoms. This is a consequence of the larger ionic radius. The substitution of the halide plays also an important role in the bond distance between the metal and the nitrogen of the pyrazine ring. This results in longer distances when moving from Cl (2.33 Å) to Br (2.55 Å) and I (2.77 Å) in the case of the Cr-based family. This can be explained by the higher covalent nature of the bond when moving down the periodic table.

**Figure 2:**
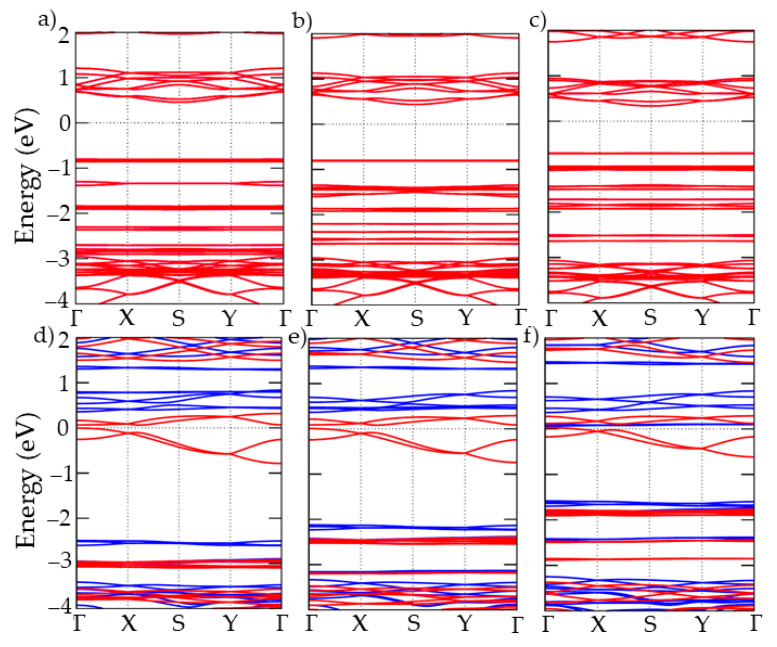
Electronic band structure of (**a**) VCl_2_(pyz)_2_, (**b**) VBr_2_(pyz)_2_, (**c**) VI_2_(pyz)_2_, (**d**) CrCl_2_(pyz)_2_, (**e**) CrBr_2_(pyz)_2_ and (**f**) CrI_2_(pyz)_2_. Blue (red) color represents states with spin up (down) in the PBE + U calculations.

Then, we calculate the electronic band structure by means of PBE + U along the high-symmetry points Γ-S-X-Y-Γ (Figure 2) of the Brillouin zone of the orthorhombic lattice [50]. We used U values of 5 and 4 eV for the V and Cr derivatives, respectively. These were extracted from the literature [18,19] and reproduce well the insulating and metallic nature of the VCl_2_(pyz)_2_ and CrCl_2_(pyz)_2_ bulk compounds, respectively (Figure S2). The density of states of the six investigated monolayers is reported in Figures S3–S8. In the case of the single-layer systems, the electronic band structures show direct bandgaps (at S high symmetry *k*-point, Figure 2) of 1.25, 1.20 and 1.02 eV for VCl_2_(pyz)_2_ (Figure 2a), VBr_2_(pyz)_2_ (Figure 2b) and VI_2_(pyz)_2_ (Figure 2c), respectively. This gives rise to insulating behavior, which is preserved either at the 2D limit or by chemical substitution. In the case of the Cr-based compounds, for CrCl_2_(pyz)_2_ (Figure 2d) and CrBr_2_(pyz)_2_ (Figure 2e), the systems turn out to be semimetallic (with the highest occupied and lowest unoccupied levels having the same spin polarization). This contrasts with the metallic nature of the CrCl_2_(pyz)_2_ bulk compound. Conversely CrI_2_(pyz)_2_ is metallic (Figure 2f) and presents two new bands above the Fermi level, which correspond to Cr *d* orbitals (Figure S8).

**Figure 3:**
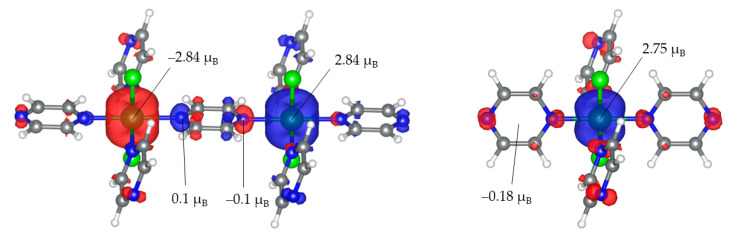
DFT-calculated spin density of a representative fragment of (**left**) VCl_2_(pyz)_2_ and (**right**) CrCl_2_(pyz)_2_. The isosurface value is set to 0.004.

In order to explore the magnetic properties of the targeted systems, we determine the magnetic exchange interactions (J) between the magnetic centers. The J values are calculated using the Gaussian09 package on a representative fragment formed by 75 and 43 atoms for VCl_2_(pyz)_2_ and CrCl_2_(pyz)_2_ (Figure 3), respectively. This allows us to compute them using the difference in energies between the high-spin state and the broken-symmetry situation. This is a standard procedure often used in quantum chemistry to study molecular magnetic materials [51]. In the case of the VX_2_(pyz)_2_ family (X = Cl, Br, I), magnetic exchange is due to the interaction of the V centers in the system because in these compounds the pyrazine ligand is not reduced to a radical. From DFT, we calculate J = −40.57 cm^−1^ in the VCl_2_(pyz)_2_ system (within the −2*J*∑_ij_*S_i_S_j_* Hamiltonian definition). This is compatible with previously reported J values for the bulk [18,19]. The broken-symmetry configuration for two V(II) magnetic centers results in *S* = 3/2 − *S’* = −3/2. This provides magnetic moment values of 2.84 μ_B_ and −2.84 μ_B_ for each V(II) center. As expected, one can observe that the neutral bridge pyrazine holds a negligible spin density, and thus there is superexchange coupling mediated by the pyrazine ring. This results in a moderate change of J values, as shown in Table 1, due to the slight structural changes reported in Table S1.

Conversely, in the case of the CrX_2_(pyz)_2_ family (where X = Cl, Br, I), the magnetic intralayer interactions take place between each Cr(III) metal center (*S* = 3/2) and the pyrazine radicals (*S* = 1/2). According to our calculations, this scenario gives rise to a final ferrimagnetic state with an isotropic J value of −2034.13 cm^−1^, which is ~50 times higher than that of CrCl_2_(pyz)_2_. We obtain spin moments of 2.75 μ_B_ on Cr(III) and −0.72 μ_B_ on the pyrazine ligands, which are in good agreement with those expected for a broken-symmetry case *S* = 3/2 − *S’* = −1/2. The total spin density on the pyrazine radicals is mostly due to the local magnetic moments of the N atoms (−0.18 for each pyrazine) and a small contribution of the magnetic moments of C, while the local magnetic moments of Cl are almost negligible. Upon halogen substitution, the magnetic exchange coupling substantially decreases by 27.2% for CrBr_2_(pyz)_2_ and by a drastic change of 72.9% for CrI_2_(pyz)_2_ (Table 1). This is different from the VX_2_(pyz)_2_ family case because the magnetic interaction mainly takes place between the Cr(III) and the radical. Concretely, the competition between a ferrimagnetic interaction Cr(III)-radical versus a ferromagnetic one between Cr^3+^ ions (t_2g_–e_g_) when decreasing the ligand field from Cl-Br-I is enhanced. This combined with the small increase in the distance between the magnetic centers (See Table S1) leads to a more noticeable reduction of the J values in CrX_2_(pyz)_2_.

Halogen substitution in the first coordination sphere has a direct effect on the ligand field around the metal. Thus, we determine the single-ion anisotropy (D) of the six compounds presented in this work. For that, we use NEVPT2 as implemented in the Orca package, which is a second-order perturbation theory using a CAS-like reference wavefunction (See Methods). In the case of the CrX_2_(pyz)_2_ series, the values of the single-ion anisotropy are 0.331 (Cl), −0.242 (Br) and −0.298 (I) cm^−1^ (E/D values are 0.217, 0.090 and 0.299, respectively). These values follow the trend experimentally observed in similar octahedral Cr compounds, where there is a significant change in the behavior of D from Cl to Br/I, due to the higher ligand field of the former [52]. This has its origin in the fact that the diffuse electron density around the nuclei of the heavier atoms causes a less effective overlap of the orbitals involved in the halide-metal bond. For the VX_2_(pyz)_2_ family, the calculated D values are 0.140 (Cl), 0.190 (Br) and 0.228 (I) cm^−1^ (E/D values are 0.195, 0.195 and 0.220, respectively). As in the previous case, the observed effect of the substitution with heavier halides is to increase the value of D, due to the enhancement in SOC in the ligands.

**Figure 4:**
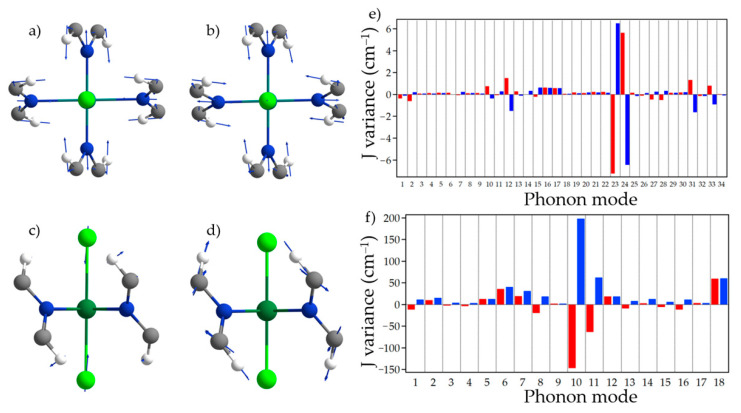
(**a**–**d**) Graphical representation of phonon mode (**a**) 23 and (**b**) 24 of VCl_2_(pyz)_2_; phonon mode (**c**) 10 and (**b**) 18 of CrCl_2_(pyz)_2_. Blue arrows represent the eigenvectors of each normal mode; (**e**,**f**) Effect of the phonon distortion on the J values for (**e**) VCl_2_(pyz)_2_ and (**f**) CrCl_2_(pyz)_2_. Color code: negative (positive) phase of oscillation in blue (red).

To investigate the effect of the lattice on the spin states, we explore spin-phonon coupling (SPC) in both families. First, we calculate the phonon normal modes in Gaussian09 using periodic boundary conditions. This allows us to generate a series of distorted coordinates following each mode until the differential energy of the distorted coordinates is equal to the next normal mode (see Section S4 in the Supplementary Material). From each finite displacement—point-by-point—calculation, we evaluate the variation in J with respect to the displacement. This is indicative of how each mode is coupled to the magnetic properties. The organic-based nature of the targeted compounds results in a unit cell that contains 23 atoms and thus leads to 66 phonon normal modes. Assuming a Boltzmann distribution, we selected the ones that have a significant population below the experimental T_C_, i.e., ~120 K and ~55 K for VCl_2_(pyz)_2_ and CrCl_2_(pyz)_2_, respectively (see Section S3 in the Supplementary Material). For each mode, we determine the J values following the set of finite distorted coordinates. This permits us to evaluate the effect of the vibrational modes in the magnetic exchange, which is indicative of SPC (Figure 4). We can observe that some modes cause a maximum variance of ~7 (VCl_2_(pyz)_2_) and 198 cm^−1^ (CrCl_2_(pyz)_2_). In the case of VCl_2_(pyz)_2_, one can observe that modes 23 and 24 cause the maximum variation for both negative and positive oscillation phases. This fact is easily explained because these lattice vibration modes directly affect V-N bond elongation and compression, both in-phase (Figure 4c and Figure S46) and out-of-phase (Figure 4d and Figure S47). Hence, they change directly the distance between the V(II) magnetic centers, thus affecting directly the nature of magnetic exchange. However, the overall effect of these modes will not be very significant because the antisymmetric character of both phases acts to partially cancel the net effect on the magnetic parameters. In CrCl_2_(pyz)_2_, the most drastic effects are observed for mode 10, which reaches almost 198 cm^−1^ of variation. This mode consists of the in-phase movement of the pyrazines towards the closer pyrazine ring. In addition, mode 18 also gives a noticeable variation in magnetic exchange due to the movement of the Cr(III) central ions towards the vicinity of two pyrazine rings in each extreme of the harmonic oscillation. This could be exploited by means of an external activation at low temperatures of specific lattice vibration modes, thus allowing an efficient tuning of the magnetic properties of the system. In addition, the opportunity of chemical substitution opens a window to change the resonant frequency of a selected lattice vibration mode to another region. We checked this possibility by calculating the vibrational lattice modes of VI_2_(pyz)_2_. In the latter, mode 24 is red-shifted from 675 cm^−1^ in VCl_2_(pyz)_2_ to 640 cm^−1^. This is due to the ratio k/m between both systems, k being the force constant and m being the reduced mass, which decreases from 0.267 to 0.24 cm^−1^.

As a next step, we investigate the thermal evolution of J and D due to the temperature-dependent population of phonon modes in CrCl_2_(pyz)_2_ as a case study. We recalculate both parameters at four finite displacements of the atomic coordinates following the eigenvectors while varying *Q_k_*, where *Q_k_* is a given real value of the distortion coordinate of the vibrational mode *k*. Then, we estimate the second derivative of the variation in these parameters with respect to *Q_k_* (See Section S5 in the Supplementary Material). We include the effect of temperature by considering a Gran Canonical Ensemble [53]. The thermal dependence of a magnetic parameter (B) is calculated as follows:

(1) $$\overset{-}{\langle B\rangle }\left(T\right)\approx \overset{-}{\langle B\rangle }\left(T=0\right)+\sum_{K=1}^{R}\left[\frac{\hbar }{4\pi }{\left(\frac{\partial^{2}B}{\partial Q_{k}^{2}}\right)}_{e}\frac{1}{m_{k}\nu_{k}}\langle n_{k}\rangle \right]$$

where $\overset{-}{\langle B\rangle }\left(T=0\right)$ and $\langle n_{k}\rangle$ are

(2) $$\overset{-}{\langle B\rangle }\left(T=0\right)\approx B_{e}+\frac{\hbar }{8\pi }\sum_{K=1}^{R}\left[{\left(\frac{\partial^{2}B}{\partial Q_{k}^{2}}\right)}_{e}\frac{1}{m_{k}\nu_{k}}\right]$$

(3) $$\langle n_{k}\rangle =\frac{1}{e^{\upsilon_{k}/k_{B}T}-1}$$

$B_{e}$ is the magnetic parameter at the undistorted geometry; $\nu_{k}$ is the frequency and $m_{k}$ is the reduced mass of the vibrational mode *k*.

**Figure 5:**
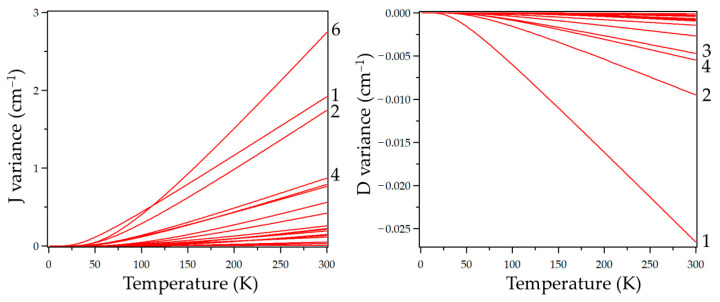
Thermal evolution of the effect on the J (**left**) and D (**right**) caused by each phonon mode (modes with higher effect are indicated at the edge of each graph).

The effect of each phonon normal mode on the thermal evolution of J and D is represented in Figure 5. We can see that J and D show the opposite behavior versus temperature. Regarding J, all phonon modes lead to an enhancement in magnetic exchange coupling. One may observe that the total effect of each mode is quite similar, except for the 6, 10 and 18 modes, which exhibit a much larger contribution (See Table S4). This explains why once mode 6 starts to be populated, its contribution becomes the most dominant. Meanwhile, modes 10 and 18 show almost a negligible contribution to the variation in J despite their enormous effect. This is a consequence of their higher activation energies, presenting a negligible effect at the selected range of temperatures. Conversely, D follows a downward trend with increasing temperature. In this case, as the individual effect of each mode is quite similar (See Table S5), the net result is governed by the vibrations that are populated at lower temperatures. These are the vibrations activated at lower frequencies, i.e., modes 1–4 (Figure 5). This validates our initial assumption, where we only consider those modes with a significant population up to the magnetic phase transition temperature. We can observe that these four vibrational modes have a prominent effect even at lower temperatures than the T_C_ value of 55 K. This underlines the importance of the study of thermal effects on such parameters as, even at low temperatures, lattice vibrations vary in a non-negligible way key parameters that govern the magnetic behavior. Overall, our results suggest that spin-phonon coupling in this family is rather weak, as the response of the magnetic properties to the phonon displacements is mostly caused by normal modes that resonate at lower frequencies. The most interesting case is that of phonon mode 6, which dominates the thermal evolution of J above 100 K. In that mode, the halide ions are moving towards the vicinity of two pyrazine rings in each phase of the oscillation (Figure S45), causing a separation of these rings which affect directly the magnetic exchange coupling between Cr and the radical. This effect would be more pronounced at lower temperatures in the case of the CrI_2_(pyz)_2_ compound because it resonates at even lower frequencies (98.9 cm^−1^) in CrI_2_(pyz)_2_) compared with 140.1 cm^−1^ in CrCl_2_(pyz)_2_, due to the heavier mass of I. As a synthetic insight to limit the effect of this phonon mode on the magnetic behavior, we suggest the introduction of steric repulsion on the pyrazine rings, e.g., by substituting H with methyl groups. This will eventually act over the effect of mode 6 by restricting the pyrazine movement, which will lead to quenching or diminishing of spin-phonon coupling through this mode.

The high synthetic versatility of MOFs represents a good opportunity to quench/enhance lattice vibrations that affect either positively or negatively the magnetic parameters. This permits the tailoring of the magnetic behavior by designing new MOF magnets with the aim of hardening/softening specific lattice vibrations. Furthermore, the described magnetic and electronic properties of the studied compounds and the prominent new applications of the novel 2D MOFs make these MX_2_(pyz)_2_ appealing candidates in research fields such as spintronics or magnonics [14,15]. Chemically, the studied compounds have a set of similarities with the well-known V(TCNE)_x_ magnetic material, which is achieving a remarkable performance in the field of magnonics [54]. This is because V(TCNE)_x_ contains several magnon modes with high quality factors, such as long lifetime and low Gilbert damping arising from the small SOC of the system. In V(TCNE)_x_ the V(II) centers are coupled with an unpaired electron lying in the C-C bond in an S = 3/2 − S’= 1/2 magnetic configuration that is quite similar to the one found in CrCl_2_(pyz)_2_. Although we expect that VCl_2_(pyz)_2_ compound will not display magnon transport due to the large distance between the V(II) magnetic centers, the magnetic exchange interaction between them could be modified by lattice-strain engineering [55], which can also be interesting for magnon straintronics applications [56]. Conversely, CrCl_2_(pyz)_2_ could be more interesting because there is antiferromagnetic coupling between Cr(III) and the delocalized radical between the pyrazine rings. So, these unpaired spins are located in a similar disposition to those of V(TCNE)_x_. Moreover, magnetic exchange interactions that take place in CrCl_2_(pyz)_2_ are in the same energy range as the calculated J in V(TCNE)_x_ [57,58]. This motivates further exploration of this type of compound toward its application in magnonics, as they could represent a great opportunity to push back the frontiers of MOFs in this emerging field.

## 4. Conclusions

We investigated the structural, electronic and magnetic properties of two appealing magnetic MOFs, namely CrCl_2_(pyz)_2_ and VCl_2_(pyz)_2_, by means of first principles at the 2D limit. Firstly, we demonstrated the feasibility of isolating a single layer, as their simulated cleavage energies are similar to those reported for paradigmatic 2D materials. Regarding their magnetic properties, our results reveal robust intralayer magnetic exchange interactions, reaching −2034.13 and −40.57 cm^−1^ for the Cr and V derivatives, respectively. Then, we simulate the effect of halogen substitution by Br and I on the properties, which results in a decrease in J, due to the lengthening of the bonds in the systems, and an enhancement of single-ion anisotropy, due to the larger SOC of the heavier halides. Finally, we perform a full analysis of spin-phonon coupling in CrCl_2_(pyz)_2_ and VCl_2_(pyz)_2_ by applying a finite displacement approach. This allowed us to unveil the most coupled phonon modes and their thermal evolution. Overall, we observed modest spin-phonon coupling in this class of materials, which is consistent with their excellent magnetic performance. Our results pave the way to their improvement by effective manipulation of the phonon modes that have a major impact on the magnetic properties of this family, e.g., moving them towards other resonant frequencies where they may be easily activated or triggered by external stimuli.

## Figures and Tables

**Table 1:** Calculated magnetic exchange constants of each studied compound in cm^−1^.

	J (cm^−1^)	
X	VX_2_(pyz)_2_	CrX_2_(pyz)_2_
Cl	−40.57	−2034.13
Br	−37.02	−1480.83
I	−34.43	−549.66

## Supplementary Materials

The following supporting information can be downloaded at: https://www.mdpi.com/article/10.3390/nano13071172/s1, Table S1: Geometrical parameters of the VX_2_(pyz)_2_ and CrX_2_(pyz)_2_ systems as described in Figure S1. Figure S1: Structural scheme followed to define the structural parameters of the described systems. Color code: M (dark green), Cl (green), C (black), N (blue) and H (pink). Figure S2: Electronic band structure of (left) CrCl_2_(pyz)_2_ and (right) VCl_2_(pyz)_2_ bulk materials. Figure S3: Calculated projected density of states (pDOS) for VCl_2_(pyz)_2_. Figure S4: Calculated projected density of states (pDOS) for VBr_2_(pyz)_2_. Figure S5: Calculated projected density of states (pDOS) for VI_2_(pyz)_2_. Figure S6: Calculated projected density of states (pDOS) for CrCl_2_(pyz)_2_. Figure S7: Calculated projected density of states (pDOS) for CrBr_2_(pyz)_2_. Figure S8: Calculated projected density of states (pDOS) for CrI_2_(pyz)_2_. Table S2: Population analysis parameter for each phonon mode in VCl_2_(pyz)_2_ at 120 K. Table S3: Population analysis parameter for each phonon mode in CrCl_2_(pyz)_2_ at 55 K. Figures S9–S26: Calculated J values as a function of the distorted geometries Q_k_ with the fitting 2nd grade polynomial for phonon modes 1–18 at the CrCl_2_(pyz)_2_ system. Table S4: ${\left(\frac{\partial^{2}J}{\partial Q_{k}^{2}}\right)}_{e}$ calculated terms and Q_k_ for each phonon mode in CrCl_2_(pyz)_2_ system. Figures S27–S44: Calculated D values as a function of the distorted geometries Q_k_ with the fitting 2nd grade polynomial for phonon modes 1–18 at the CrCl_2_(pyz)_2_ system. Table S5: ${\left(\frac{\partial^{2}D}{\partial Q_{k}^{2}}\right)}_{e}$ calculated terms and Q_k_ for each phonon mode in CrCl_2_(pyz)_2_ system. Figures S45–S47: Graphical representation of phonon modes 6,10 and 18 on CrCl_2_(pyz)_2_ (a) top view of negative phase (b) top view of positive phase (c) side view of negative phase (d) side view of positive phase.
