# Supplementary material for: Exploring Spin-Phonon Coupling in Magnetic 2D Metal-Organic Frameworks
Source: Nanomaterials (Basel). 2023 Mar 25;13(7):1172. doi: 10.3390/nano13071172 (PMC10097403; doi:10.3390/nano13071172)
Supplement: Supplementary file 1 [file nanomaterials-13-01172-s001.zip › nanomaterials-2305676-supplementary.pdf]

Supporting Information

Exploring Spin-Phonon Coupling in Magnetic 2D Metal-Organic Frameworks

Diego López-Alcalá, Alberto M. Ruiz and José J. Baldoví *

Instituto de Ciencia Molecular, Universitat de València, 46980 Paterna, Spain
* Correspondence: j.jaime.baldovi@uv.es

S1. Structural Parameters

Table S1. Geometrical parameters of the $VX_2(pyz)_2$ and $CrX_2(pyz)_2$ systems as described in Figure S1.

X	M = Cr	M = V
**Cl**		
α (°)	25.68	25.89
β (°)	90.42	90.12
γ (°)	123.68	123.19
Cl-M (Å)	2.33	2.38
M-N(Å)	2.09	2.15
N-C(Å)	1.36	1.36
C-C(Å)	1.37	1.38
Lattice vector a (Å)	7.049	7.156
Lattice vector b (Å)	7.047	7.156
**Br**		
α (°)	30.44	30.20
β (°)	90.52	90.17
γ (°)	124.02	123.68
Br-M (Å)	2.52	2.57
M-N(Å)	2.09	2.16
N-C(Å)	1.36	1.35
C-C(Å)	1.37	1.38
Lattice vector a (Å)	7.049	7.168
Lattice vector b (Å)	7.048	7.168
**I**		
α (°)	35.67	35.32
β (°)	90.46	90.27
γ (°)	124.89	124.14
I-M (Å)	2.77	2.82
M-N(Å)	2.10	2.17
N-C(Å)	1.36	1.35
C-C(Å)	1.37	1.38
Lattice vector a (Å)	7.084	7.210
Lattice vector b (Å)	7.081	7.210

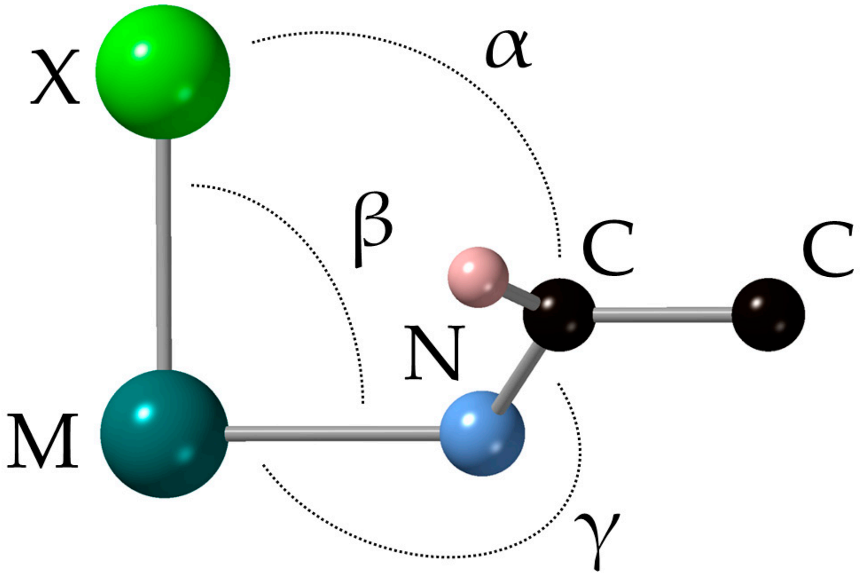

Figure S1: Structural scheme followed to define the structural parameters of the described systems. Color code: M (dark green), Cl (green), C (black), N (blue) and H (pink).

S2. Electronic structure

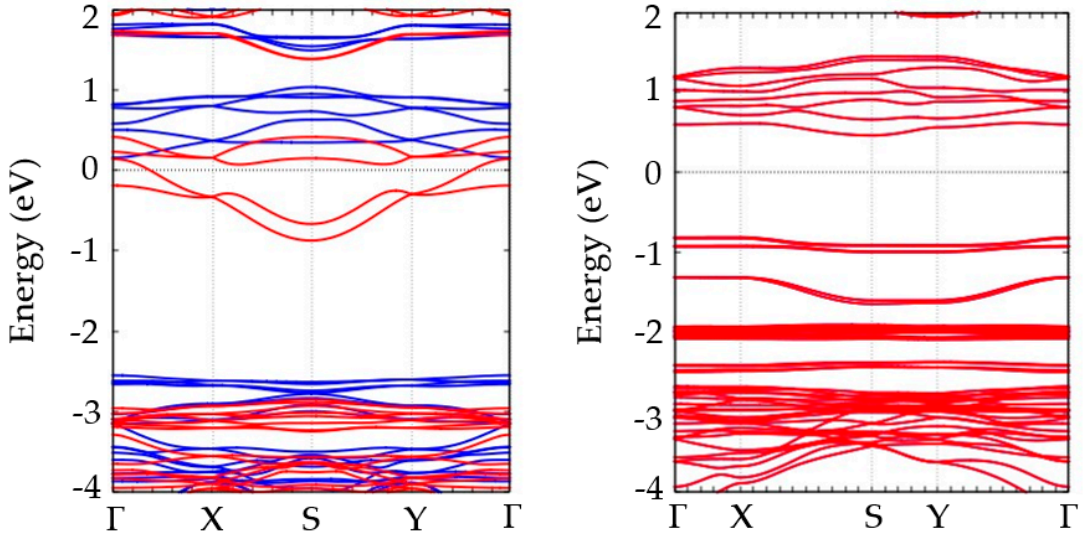

Figure S2: Electronic band structure of (left) $CrCl_2(pyz)_2$ and (right) $VCl_2(pyz)_2$ bulk materials.

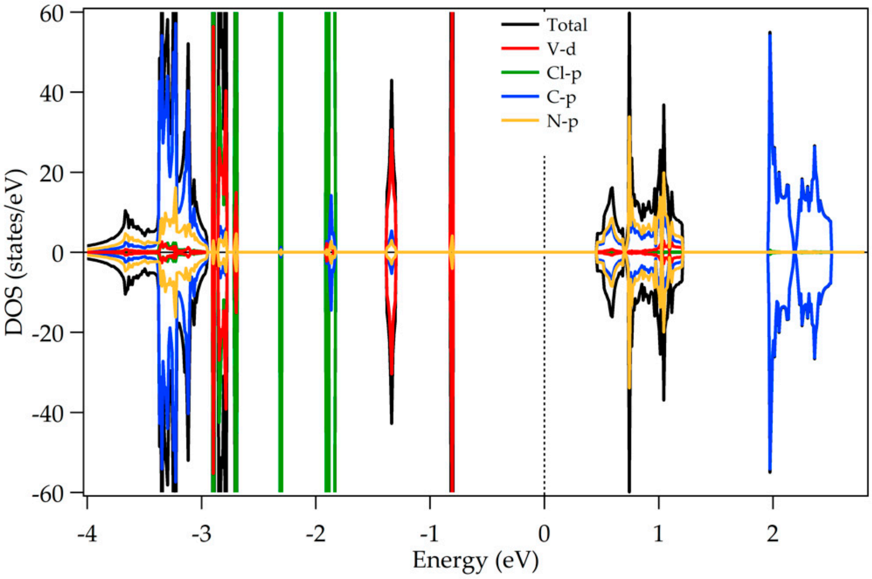

Figure S3: Calculated projected density of states (pDOS) for $VCl_2(pyz)_2$.

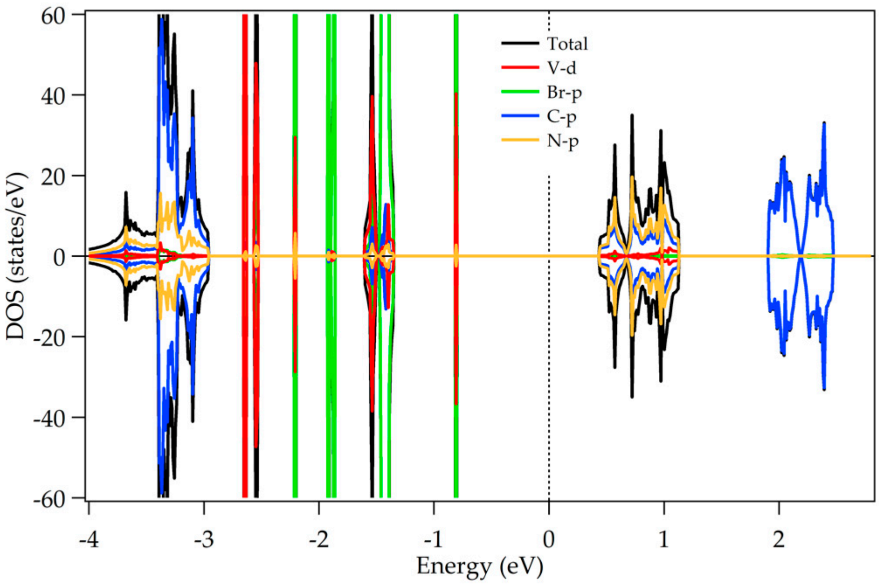

Figure S4: Calculated projected density of states (pDOS) for $VBr_2(pyz)_2$.

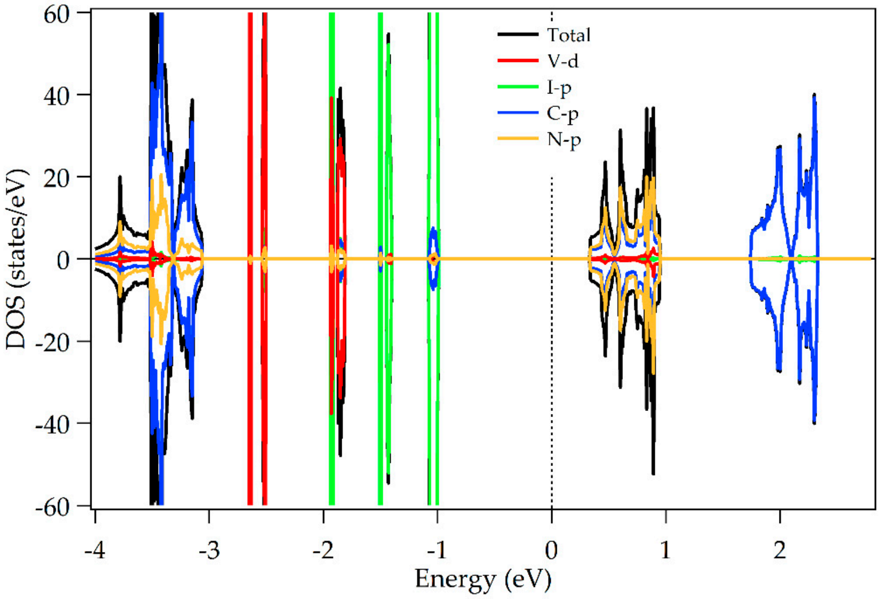

Figure S5: Calculated projected density of states (pDOS) for $VI_2(pyz)_2$.

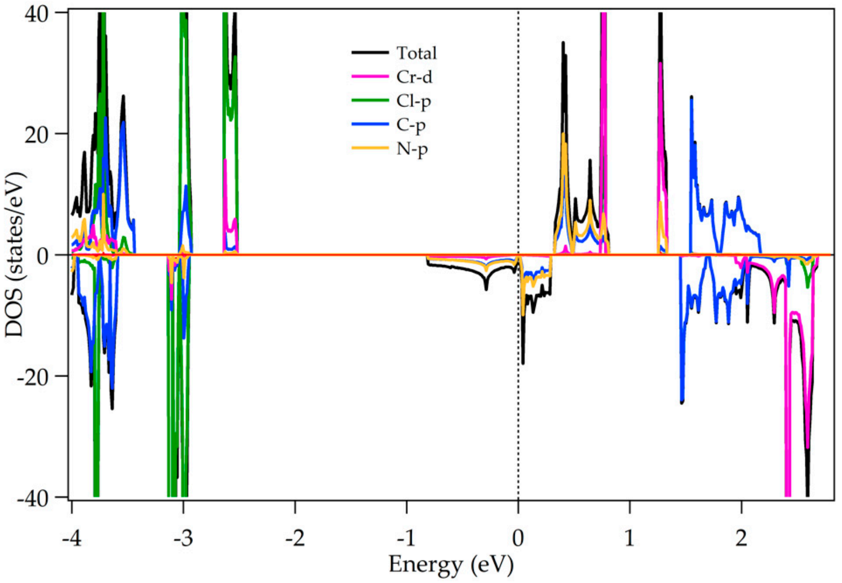

Figure S6: Calculated projected density of states (pDOS) for $CrCl_2(pyz)_2$.

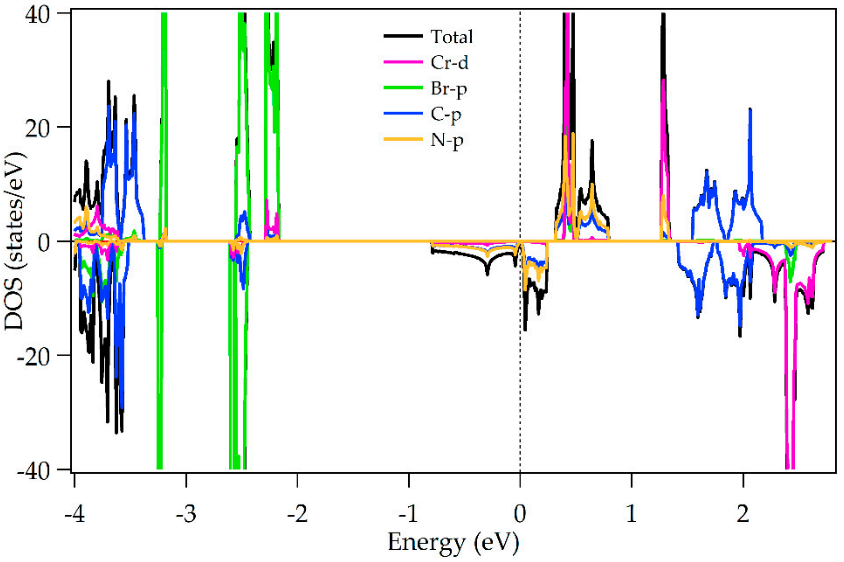

Figure S7: Calculated projected density of states (pDOS) for $CrBr_2(pyz)_2$.

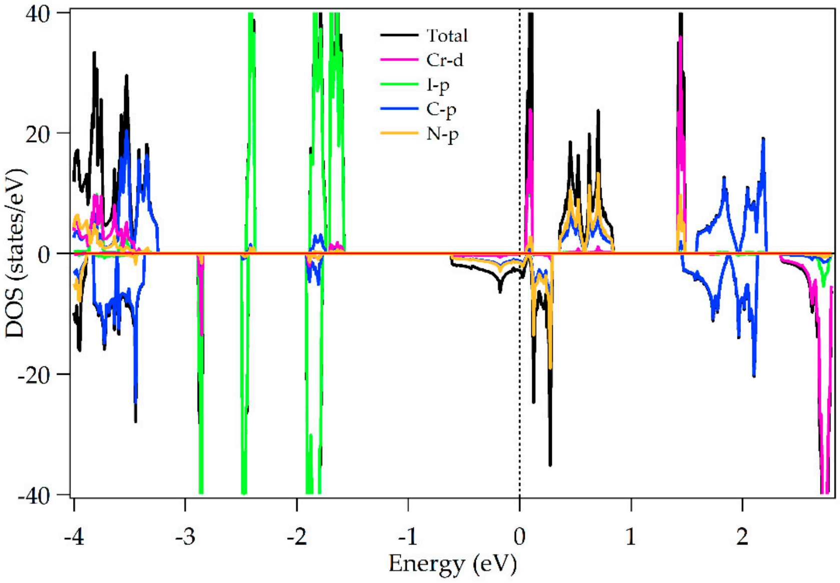

Figure S8: Calculated projected density of states (pDOS) for $CrI_2(pyz)_2$.

S3. Population of Phonon Modes

In order to elucidate the population of the phonon modes at the magnetic phase transition temperature for the $CrCl_2(pyz)_2$ and $VCl_2(pyz)_2$ compounds claimed by Pedersen et al. we assumed a Boltzmann distribution of the population of the different phonon modes. In that way, we are able to predict the probability of each vibrational state as a function of the energy and the temperature of the system as:

$$p_i = \frac{e^{-\frac{\varepsilon_i}{k_B T}}}{\sum_{j=1}^{M} e^{-\frac{\varepsilon_j}{k_B T}}} \quad \text{(S1)}$$

were p_i is the occupational probability of each state, ε_i the energy of each state and k_B the Boltzmann constant.

Then we evaluated the probabilities of occupation of each state, and we obtained the distribution of populations at the magnetic phase transition temperature of each system using the data represented in Tables S2 and S3. We considered occupied those quantity of consecutive levels which suppose a significant population of 0.9999 out of 1, considering negligible the population of the rest of the vibrational states. Considering 55 K the magnetic phase transition temperature for the $CrCl_2(pyz)_2$ system we consider significantly populated the first 18 phonon modes, with a sum of populations of 0.999903. In the case of the $VCl_2(pyz)_2$ system, with a magnetic phase transition temperature of 120 K, we consider significantly populated the first 34 phonon modes, with a sum of populations of 0.999924.

Table S2: Population analysis parameter for each phonon mode in $VCl_2(pyz)_2$ at 120 K.

Phonon mode	Frequency (cm^{-1})	Energy (eV)	Population
1	71.4201	0.008854968	0.342064157
2	107.4374	0.013320546	0.133326426
3	116.9119	0.014495235	0.104058309
4	117.9587	0.014625022	0.101247453
5	123.9167	0.01536372	0.086635417
6	126.3295	0.015662869	0.081336172
7	131.0057	0.016242644	0.071971007
8	185.1015	0.022949672	0.017481433
9	189.321	0.023472823	0.015654508
10	196.2156	0.024327645	0.013071039
11	196.6909	0.024386575	0.012909524
12	226.8012	0.028119778	0.005872569
13	235.7826	0.02923333	0.004642915
14	236.9666	0.029380127	0.004501315
15	258.4101	0.032038784	0.002568742
16	287.6041	0.03565838	0.001196874
17	289.4691	0.035889611	0.001139883
18	352.3664	0.043687886	0.000219931
19	402.3011	0.049879002	5.95634E-05
20	418.8887	0.051935603	3.85948E-05
21	524.9681	0.065087779	2.40638E-06
22	539.8906	0.066937934	1.62866E-06
23	667.9633	0.082816932	5.71216E-08
24	675.5488	0.083757415	4.68405E-08
25	724.9607	0.089883712	1.28604E-08
26	729.0079	0.090385501	1.15684E-08
27	780.4615	0.096764938	3.01101E-09
28	791.1208	0.098086523	2.27831E-09
29	860.1209	0.106641449	3.74718E-10
30	879.3985	0.109031568	2.26304E-10
31	940.3838	0.116592785	4.59029E-11
32	959.0167	0.118902971	2.81937E-11
33	1021.3622	0.126632831	5.51884E-12
34	1026.9278	0.127322878	4.77108E-12
35	1034.3347	0.128241217	3.93068E-12
36	1036.4653	0.128505378	3.71759E-12
37	1040.0627	0.128951399	3.3837E-12
38	1049.0819	0.130069638	2.67254E-12
39	1076.8711	0.133515061	1.29185E-12
40	1082.6237	0.134228293	1.11137E-12
41	1126.9455	0.139723498	3.48593E-13
42	1146.6815	0.142170451	2.08017E-13
43	1148.2565	0.142365727	1.99621E-13
44	1154.6727	0.143161234	1.68776E-13
45	1262.575	0.156539421	1.00331E-14
46	1263.2674	0.156625267	9.85305E-15
47	1273.3981	0.157881315	7.5592E-15
48	1293.0528	0.160318188	4.52043E-15
49	1390.5815	0.172410211	3.525E-16

50	1397.9189	0.173319933	2.90937E-16
51	1464.3111	0.181551521	5.12295E-17
52	1471.8633	0.182487875	4.20455E-17
53	1521.2386	0.188609634	1.1555E-17
54	1527.5701	0.189394641	9.79121E-18
55	1541.7021	0.191146786	6.76524E-18
56	1544.2819	0.191466641	6.32375E-18
57	1643.095	0.203717909	4.76828E-19
58	1657.789	0.205539734	3.24656E-19
59	3244.9538	0.402323179	3.01785E-37
60	3246.0034	0.402453313	2.93612E-37
61	3249.3426	0.402867322	2.69053E-37
62	3250.2001	0.402973638	2.63085E-37
63	3254.1586	0.40346443	2.37204E-37
64	3261.0224	0.404315433	1.98218E-37
65	3265.2646	0.404841399	1.77398E-37
66	3272.1905	0.405700101	1.48E-37

Table S3: Population analysis parameter for each phonon mode in $CrCl_2(pyz)_2$ at 55 K.

Phonon mode	Frequency (cm^{-1})	Energy (eV)	Population
1	72.129	0.00894286	0.418251995
2	122.7602	0.015220332	0.111229162
3	123.3048	0.015287854	0.109655769
4	129.3375	0.016035814	0.093647068
5	139.4894	0.017294489	0.071805678
6	140.1019	0.01737043	0.070664322
7	147.2891	0.018261529	0.058552664
8	200.8281	0.024899522	0.014430849
9	203.0703	0.02517752	0.013608753
10	217.6333	0.026983103	0.009297551
11	218.3125	0.027067313	0.009133815
12	221.2336	0.027429483	0.008461857
13	257.8686	0.031971646	0.003245314
14	260.0574	0.032243023	0.003064713
15	270.8122	0.033576449	0.002313156
16	297.5659	0.036893486	0.001148833
17	301.6344	0.037397916	0.001032844
18	342.0229	0.042405454	0.000359073
19	410.5548	0.050902331	5.97852E-05
20	432.841	0.053665469	3.33735E-05
21	535.3846	0.066379261	2.28248E-06
22	569.3302	0.070587981	9.39187E-07
23	655.9863	0.081331973	9.73318E-08
24	673.7412	0.083533301	6.11705E-08
25	721.489	0.089453276	1.75421E-08
26	727.1465	0.090154717	1.51289E-08
27	763.8723	0.094708138	5.7885E-09
28	778.2382	0.096489283	3.97517E-09
29	852.3681	0.105680224	5.717E-10
30	879.0154	0.10898407	2.84727E-10
31	911.4562	0.113006218	1.21862E-10
32	933.9501	0.115795107	6.76578E-11
33	1005.266	0.124637156	1.04737E-11
34	1009.2372	0.125129522	9.44025E-12
35	1010.9943	0.125347375	9.01615E-12
36	1016.5359	0.126034446	7.79943E-12
37	1024.2918	0.126996056	6.3672E-12
38	1032.2858	0.127987186	5.1657E-12
39	1058.0405	0.131180363	2.63348E-12
40	1063.7678	0.131890458	2.26706E-12
41	1106.6346	0.137205266	7.38677E-13
42	1122.9799	0.139231826	4.81678E-13
43	1125.2914	0.139518416	4.53415E-13
44	1131.2531	0.140257573	3.8794E-13
45	1240.3367	0.153782222	2.23599E-14
46	1243.003	0.154112801	2.08535E-14
47	1272.4675	0.157765935	9.6479E-15
48	1294.4944	0.160496924	5.42233E-15
49	1380.4206	0.17115042	5.72771E-16

50	1386.8273	0.17194475	4.8439E-16
51	1460.36	0.181061647	7.07608E-17
52	1462.8642	0.181372128	6.62739E-17
53	1488.743	0.184580692	3.36771E-17
54	1494.4162	0.185284079	2.90323E-17
55	1514.6954	0.187798381	1.70801E-17
56	1519.7883	0.18842982	1.49496E-17
57	1613.6963	0.200072934	1.28157E-18
58	1635.6234	0.202791549	7.22156E-19
59	3240.4529	0.401765139	4.2288E-37
60	3244.8503	0.402310347	3.76928E-37
61	3246.958	0.402571669	3.56708E-37
62	3251.4689	0.403130949	3.17004E-37
63	3253.5603	0.40339025	3.00127E-37
64	3259.0726	0.404073688	2.59824E-37
65	3265.2951	0.40484518	2.20793E-37
66	3274.0415	0.405929596	1.75638E-37

S4. Finite Displacements in Phonon Modes Distortion

In order to recompute the single ion anisotropy (D) or magnetic exchange coupling (J) we need to obtain the distorted geometries provoked by the phonon mode as:

$$Q_v = Q_{eq} + v \cdot Q_p \qquad \text{(S2)}$$

were Q_v is the distorted geometry, Q_{eq} is the equilibrium geometry, v is the applied finite distortion and Q_p is the vector displacement of each mode.

We consider this v as the one which equally the energy of this phonon mode with the energy of the next phonon mode, as defined by the harmonic oscillator approximation:

$$\frac{1}{2}kv^2 = \frac{1}{2}\hbar\omega \qquad \text{(S3)}$$

were k is the force constant of each phonon mode, ħ is the Planck constant and ω is the angular frequency.

Using this approximation, we applied the calculated finite displacement to each distorted geometry and then we recalculated the magnetic parameters on this situation.

S5. Calculated Variation of J and D with Q_k

For calculating the evolution of J and D as a function of the temperature we needed to evaluate the variation of these parameters within a finite change in the molecular coordinates (Q_k). We defined this Q_k as the change in each cartesian coordinate of the system within a distortion caused by a lattice vibration and we computed it as

$$Q_k = \sqrt{(x_i' - x_i)^2 + (y_i' - y_i)^2 + (z_i' - z_i)^2 \ldots} \qquad \text{(S4)}$$

were x_i' is the x Cartesian coordinate of an atom i within a phonon mode distortion and x_i is the x Cartesian coordinate of an atom i in the equilibrium position.

Then, we represented the actual value of J and D at each Q_k in order to evaluate the $\left(\frac{\partial^2 B}{\partial Q_k^2}\right)_e$ term in Equation 1 and 2 in the main text for each phonon mode (Figures S9-44) and the obtained results are summarized in Tables S4 and S5.

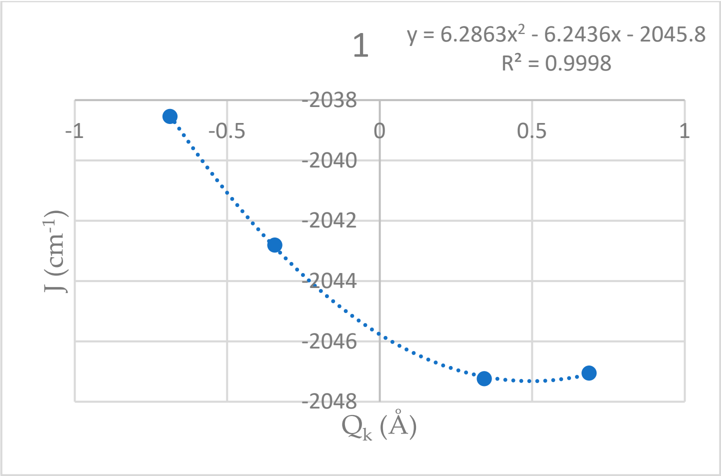

Figure S9: Calculated J values as a function of the distorted geometries Q_k with the fitting 2nd grade polynomial for phonon mode 1 at the $CrCl_2(pyz)_2$ system.

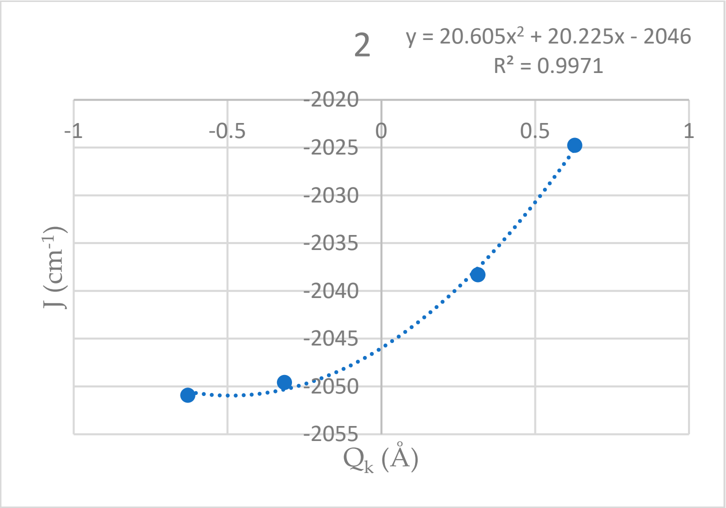

Figure S10: Calculated J values as a function of the distorted geometries Q_k with the fitting 2nd grade polynomial for phonon mode 2 at the $CrCl_2(pyz)_2$ system.

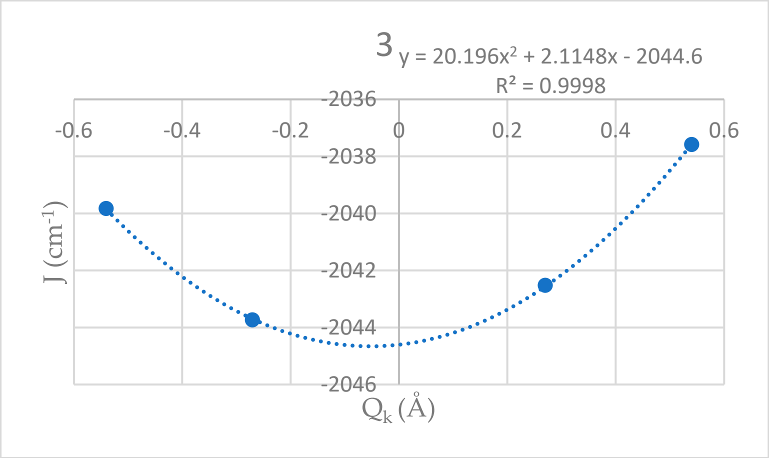

Figure S11: Calculated J values as a function of the distorted geometries Q_k with the fitting 2nd grade polynomial for phonon mode 3 at the $CrCl_2(pyz)_2$ system.

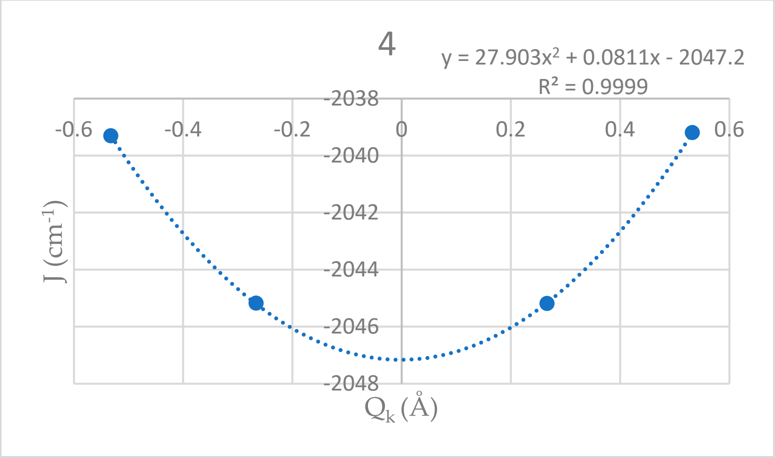

Figure S12: Calculated J values as a function of the distorted geometries Q_k with the fitting 2nd grade polynomial for phonon mode 4 at the $CrCl_2(pyz)_2$ system.

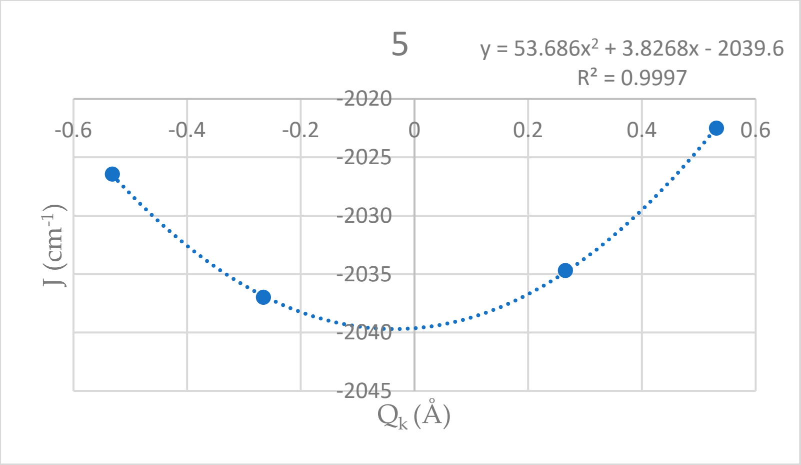

Figure S13: Calculated J values as a function of the distorted geometries Q_k with the fitting 2nd grade polynomial for phonon mode 5 at the $CrCl_2(pyz)_2$ system.

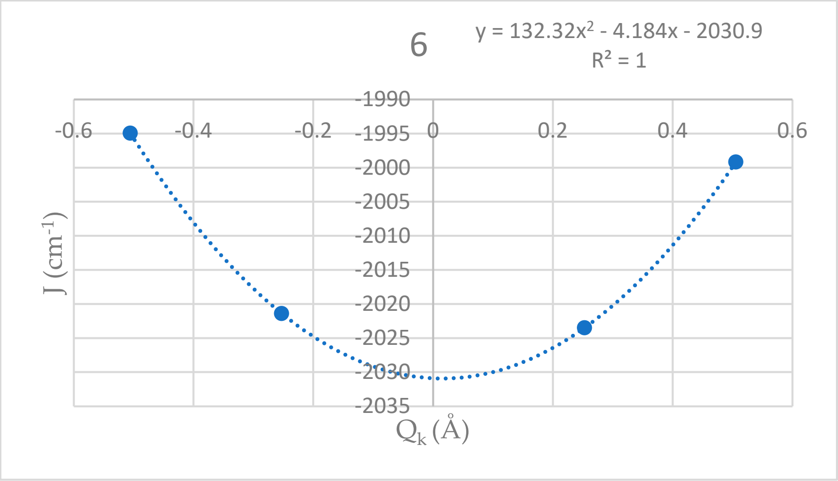

Figure S14: Calculated J values as a function of the distorted geometries Q_k with the fitting 2nd grade polynomial for phonon mode 6 at the $CrCl_2(pyz)_2$ system.

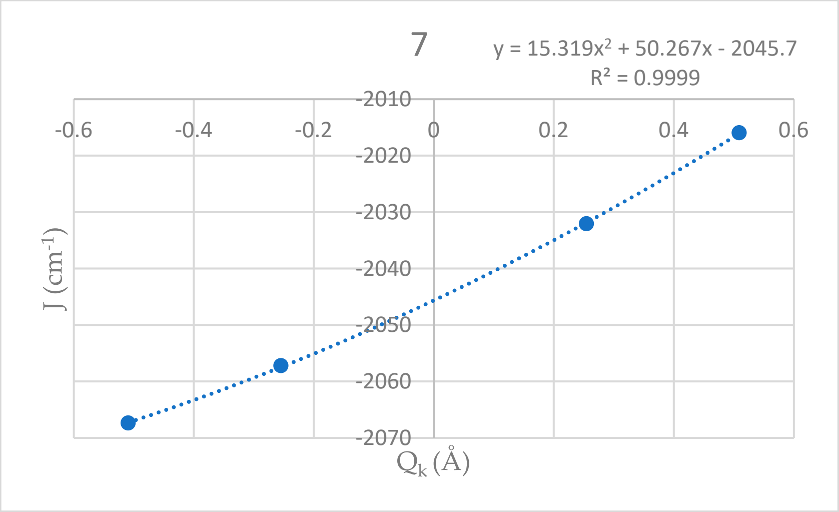

Figure S15: Calculated J values as a function of the distorted geometries Q_k with the fitting 2nd grade polynomial for phonon mode 7 at the $CrCl_2(pyz)_2$ system.

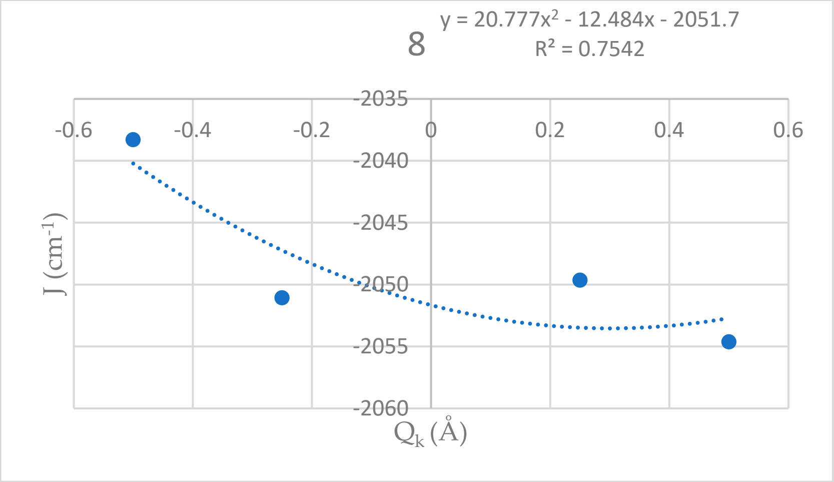

Figure S16: Calculated J values as a function of the distorted geometries Q_k with the fitting 2nd grade polynomial for phonon mode 8 at the $CrCl_2(pyz)_2$ system.

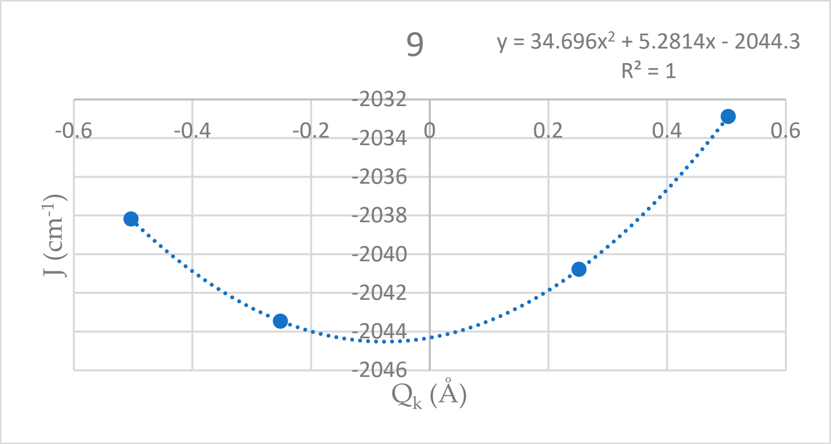

Figure S17: Calculated J values as a function of the distorted geometries Q_k with the fitting 2nd grade polynomial for phonon mode 9 at the $CrCl_2(pyz)_2$ system.

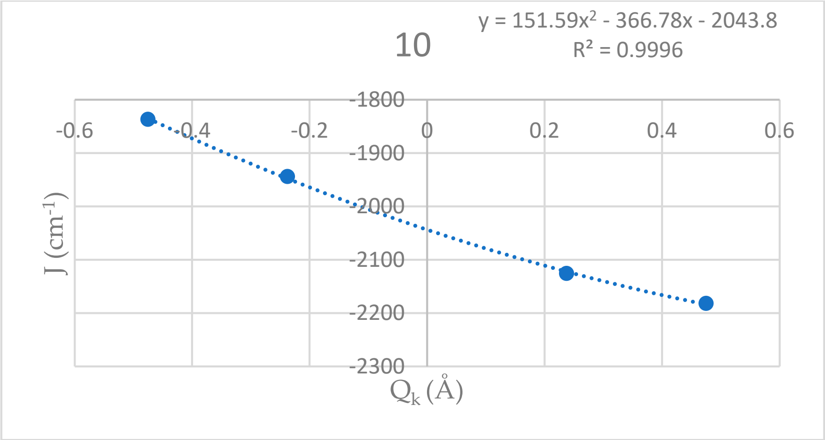

Figure S18: Calculated J values as a function of the distorted geometries Q_k with the fitting 2nd grade polynomial for phonon mode 10 at the $CrCl_2(pyz)_2$ system.

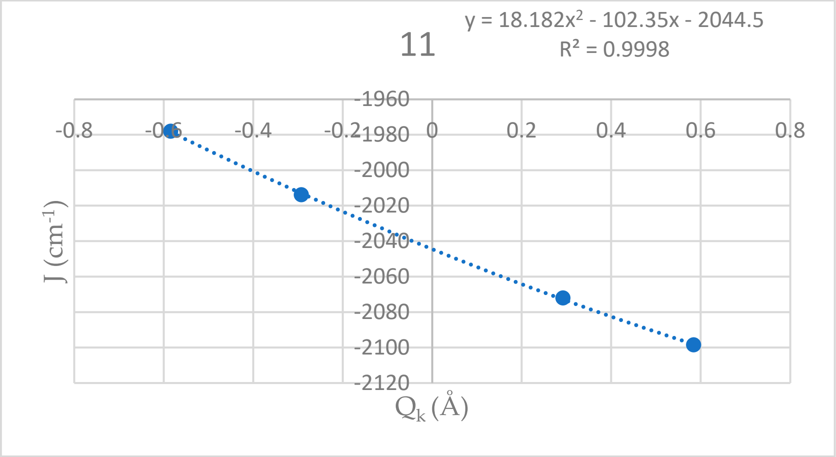

Figure S19: Calculated J values as a function of the distorted geometries Q_k with the fitting 2nd grade polynomial for phonon mode 11 at the $CrCl_2(pyz)_2$ system.

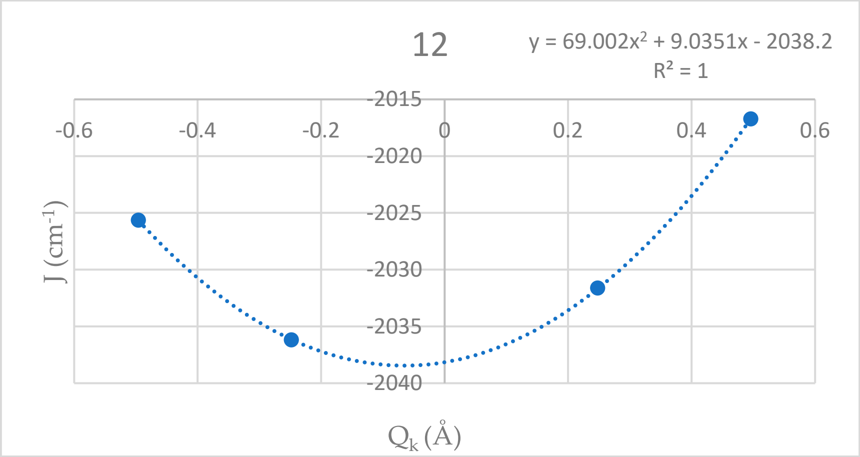

Figure S20: Calculated J values as a function of the distorted geometries Q_k with the fitting 2nd grade polynomial for phonon mode 12 at the $CrCl_2(pyz)_2$ system.

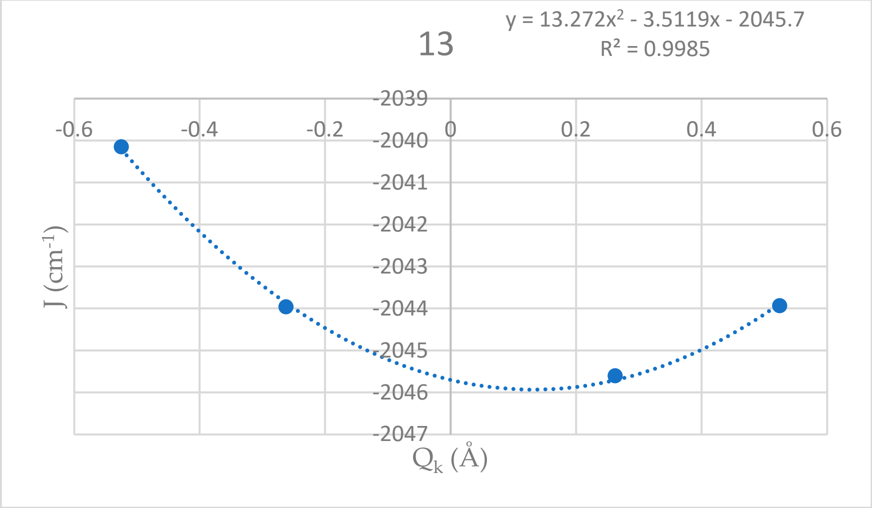

Figure S21: Calculated J values as a function of the distorted geometries Q_k with the fitting 2nd grade polynomial for phonon mode 13 at the $CrCl_2(pyz)_2$ system.

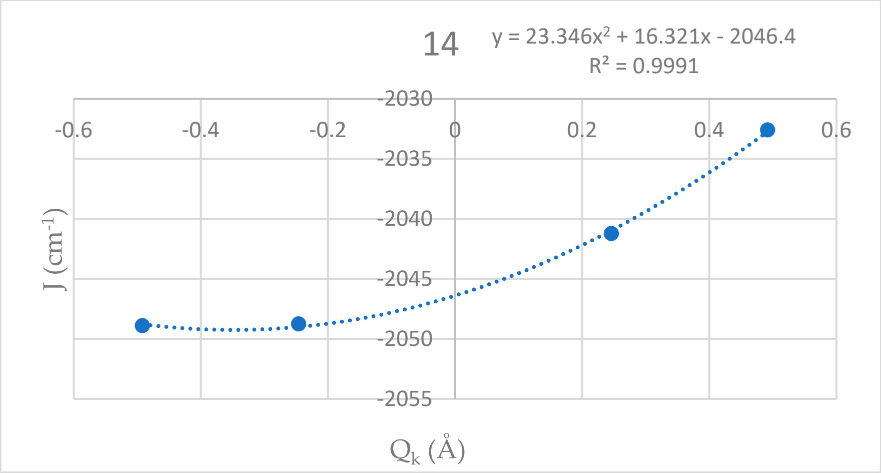

Figure S22: Calculated J values as a function of the distorted geometries Q_k with the fitting 2nd grade polynomial for phonon mode 14 at the $CrCl_2(pyz)_2$ system.

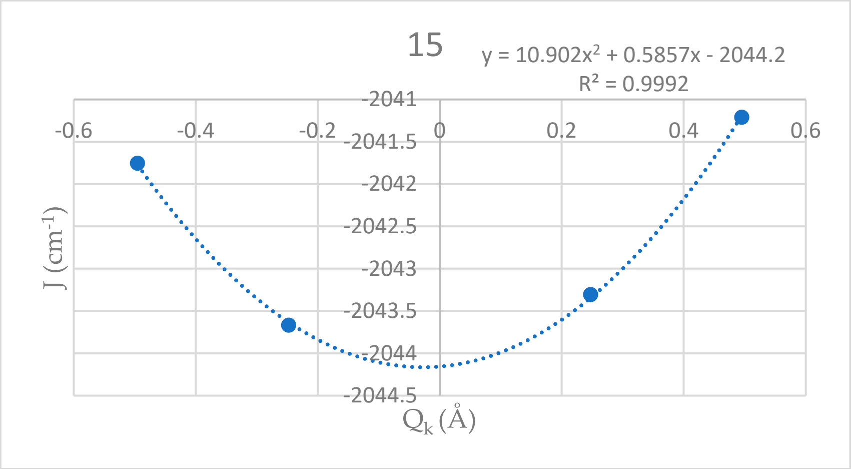

Figure S23: Calculated J values as a function of the distorted geometries Q_k with the fitting 2nd grade polynomial for phonon mode 15 at the $CrCl_2(pyz)_2$ system.

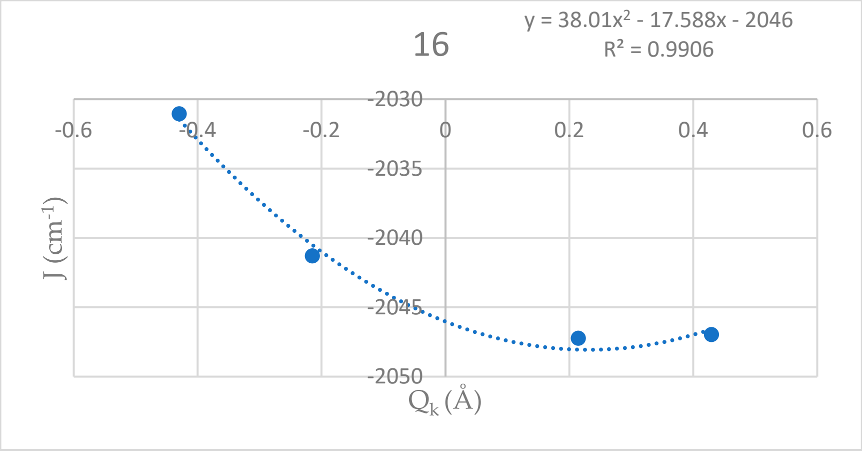

Figure S24: Calculated J values as a function of the distorted geometries Q_k with the fitting 2nd grade polynomial for phonon mode 16 at the $CrCl_2(pyz)_2$ system.

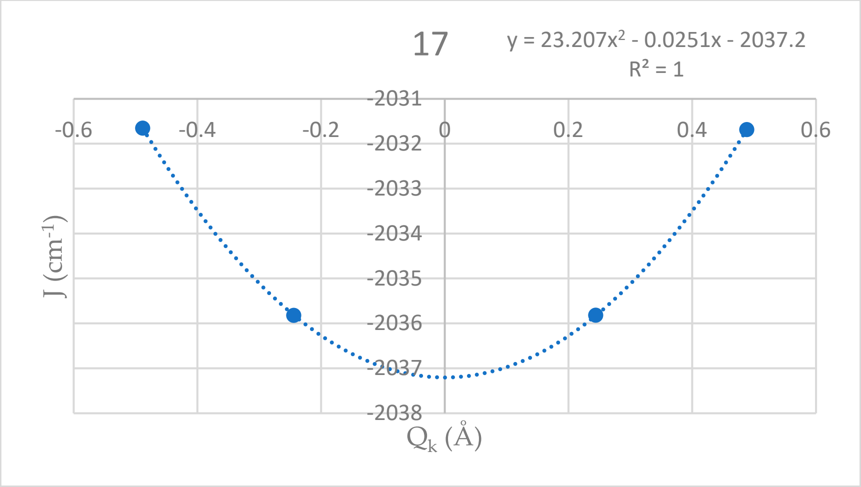

Figure S25: Calculated J values as a function of the distorted geometries Q_k with the fitting 2nd grade polynomial for phonon mode 17 at the $CrCl_2(pyz)_2$ system.

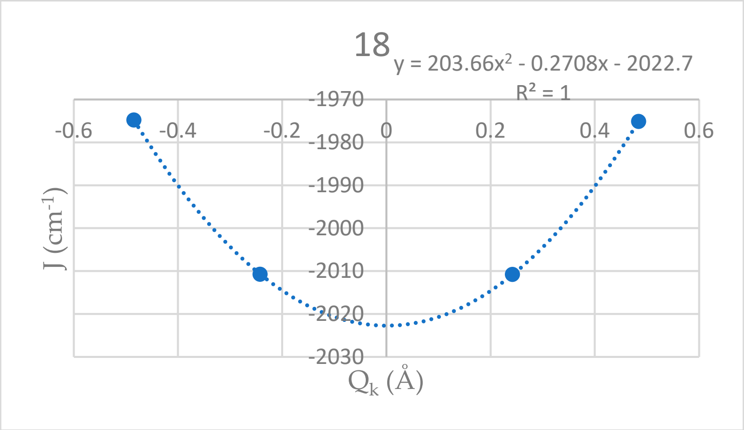

Figure S26: Calculated J values as a function of the distorted geometries Q_k with the fitting 2nd grade polynomial for phonon mode 18 at the $CrCl_2(pyz)_2$ system.

Table S4: $\left(\frac{\partial^2 J}{\partial Q_k^2}\right)_e$ calculated terms and Q_k for each phonon mode in $CrCl_2(pyz)_2$ system.

**Phonon mode**	$\left(\frac{\partial^2 J}{\partial Q_k^2}\right)_e$ **(cm-1/Å2)**	Q_k **(Å)**
1	12.57	±0.6870
2	41.21	±0.6286
3	40.39	±0.5403
4	55.81	±0.5322
5	107.37	±0.5312
6	264.64	±0.5056
7	30.64	±0.5091
8	41.55	±0.5002
9	69.39	±0.5033
10	303.18	±0.4751
11	36.36	±0.5843
12	138.00	±0.4959
13	26.54	±0.5246
14	46.69	±0.4918
15	21.80	±0.4952
16	76.02	±0.4293
17	46.41	±0.4882
18	407.32	±0.4842

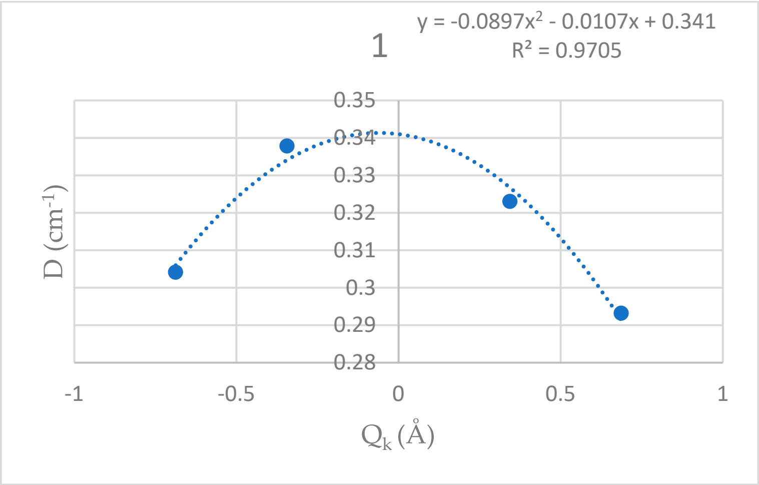

Figure S27: Calculated D values as a function of the distorted geometries Q_k with the fitting 2nd grade polynomial for phonon mode 1 at the $CrCl_2(pyz)_2$ system.

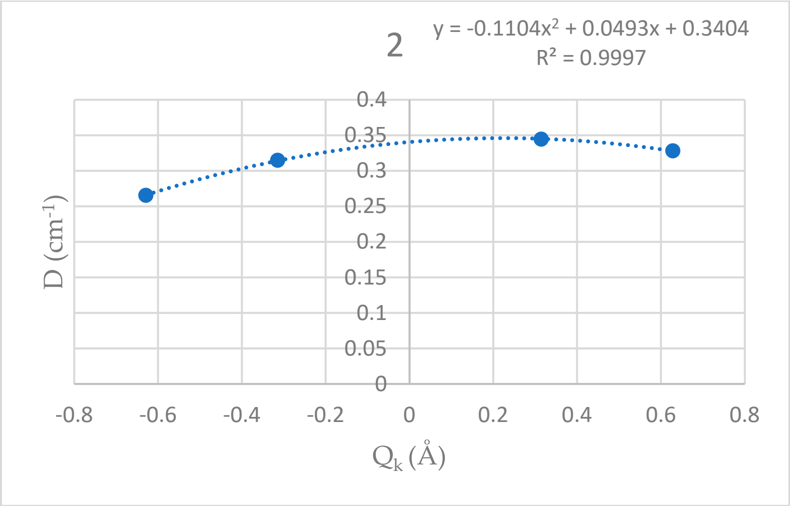

Figure S28: Calculated D values as a function of the distorted geometries Q_k with the fitting 2nd grade polynomial for phonon mode 2 at the $CrCl_2(pyz)_2$ system.

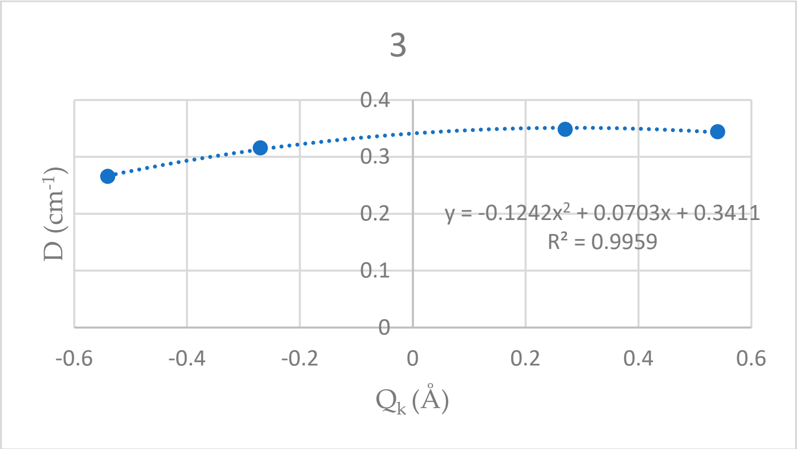

Figure S29: Calculated D values as a function of the distorted geometries Q_k with the fitting 2nd grade polynomial for phonon mode 3 at the $CrCl_2(pyz)_2$ system.

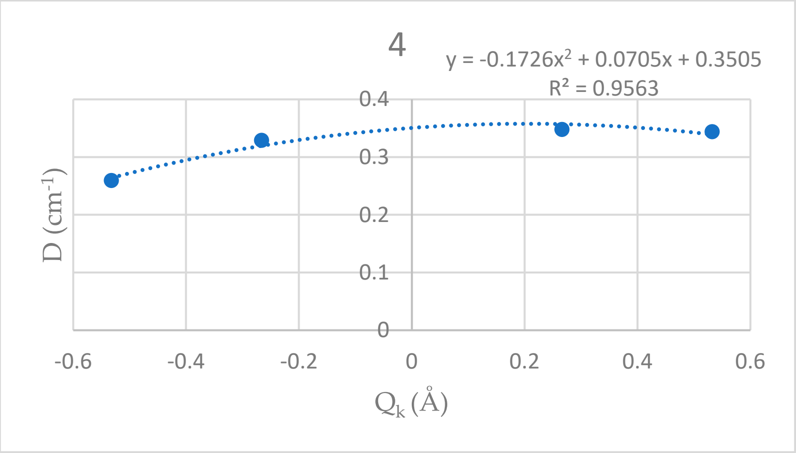

Figure S30: Calculated D values as a function of the distorted geometries Q_k with the fitting 2nd grade polynomial for phonon mode 4 at the $CrCl_2(pyz)_2$ system.

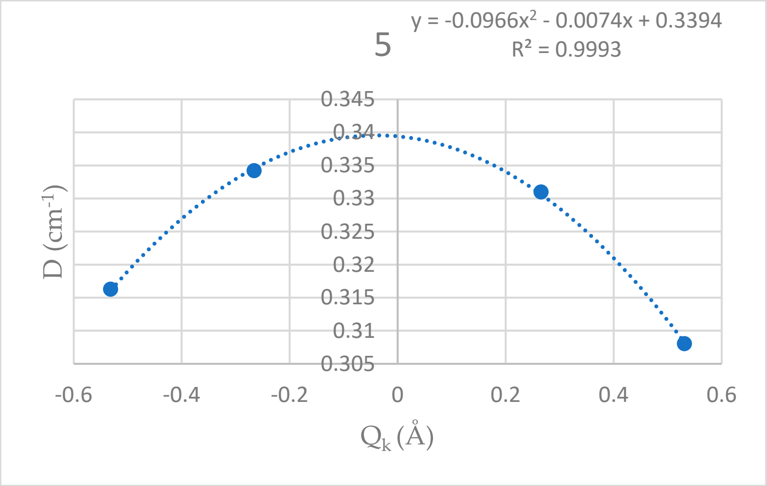

Figure S31: Calculated D values as a function of the distorted geometries Q_k with the fitting 2nd grade polynomial for phonon mode 5 at the $CrCl_2(pyz)_2$ system.

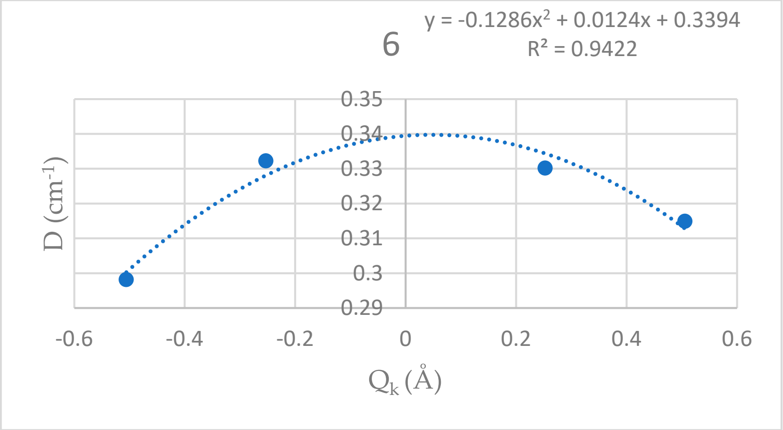

Figure S32: Calculated D values as a function of the distorted geometries Q_k with the fitting 2nd grade polynomial for phonon mode 6 at the $CrCl_2(pyz)_2$ system.

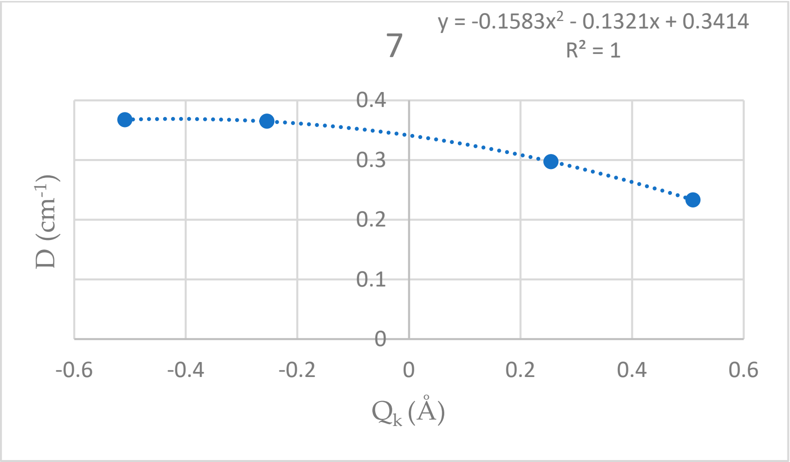

Figure S33: Calculated D values as a function of the distorted geometries Q_k with the fitting 2nd grade polynomial for phonon mode 7 at the $CrCl_2(pyz)_2$ system.

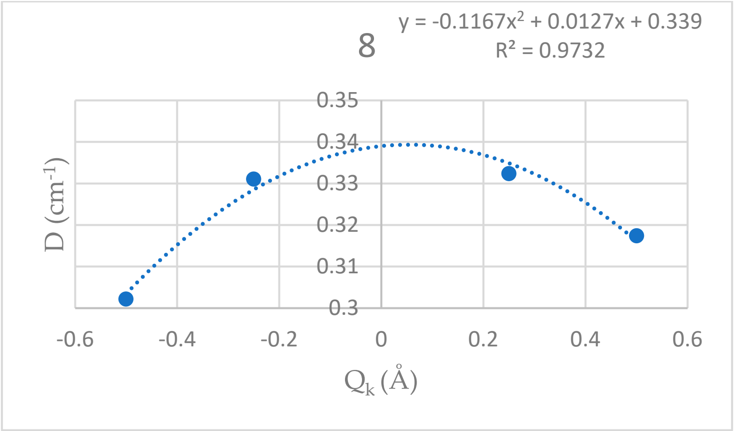

Figure S34: Calculated D values as a function of the distorted geometries Q_k with the fitting 2nd grade polynomial for phonon mode 8 at the $CrCl_2(pyz)_2$ system.

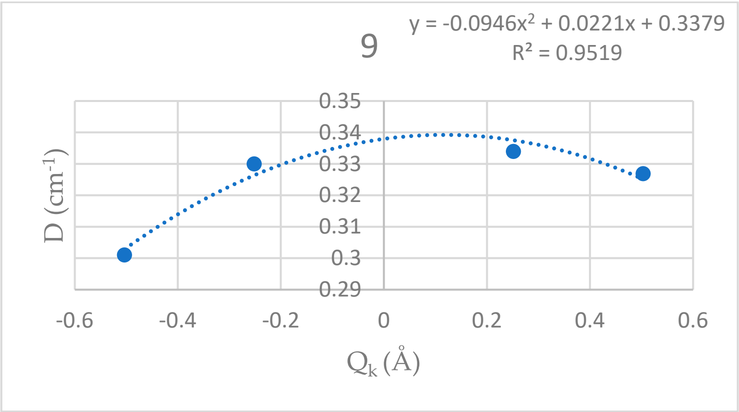

Figure S35: Calculated D values as a function of the distorted geometries Q_k with the fitting 2nd grade polynomial for phonon mode 9 at the $CrCl_2(pyz)_2$ system.

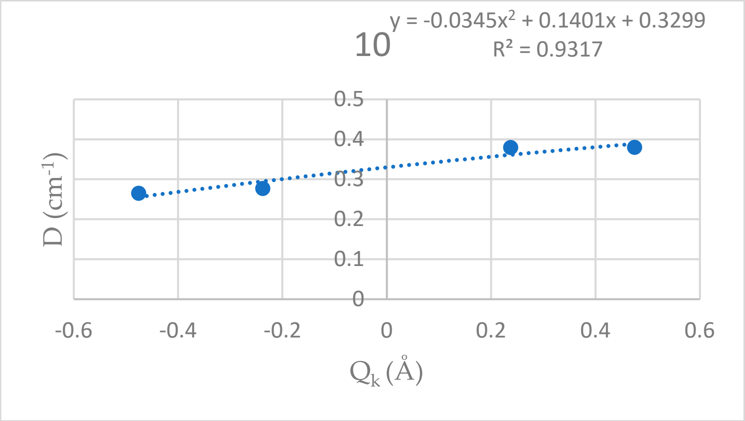

Figure S36: Calculated D values as a function of the distorted geometries Q_k with the fitting 2nd grade polynomial for phonon mode 10 at the $CrCl_2(pyz)_2$ system.

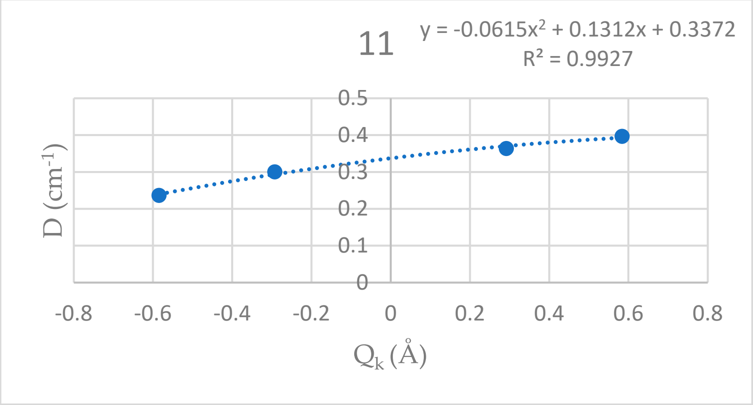

Figure S37: Calculated D values as a function of the distorted geometries Q_k with the fitting 2nd grade polynomial for phonon mode 11 at the $CrCl_2(pyz)_2$ system.

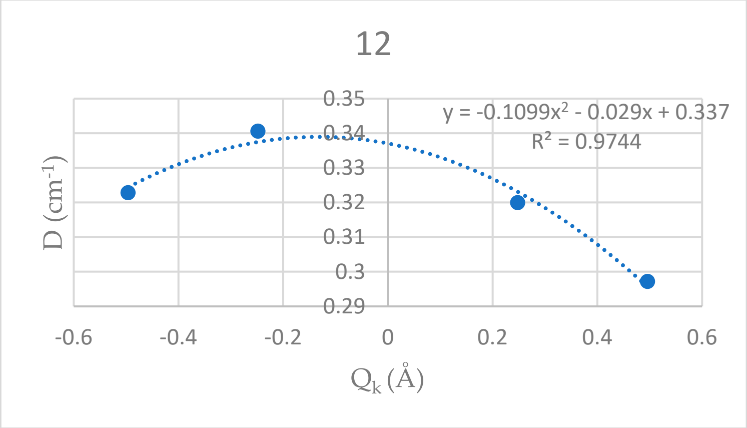

Figure S38: Calculated D values as a function of the distorted geometries Q_k with the fitting 2nd grade polynomial for phonon mode 12 at the $CrCl_2(pyz)_2$ system.

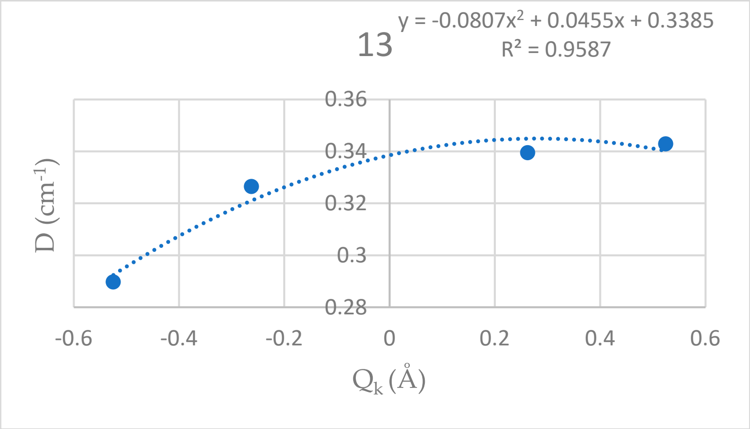

Figure S39: Calculated D values as a function of the distorted geometries Q_k with the fitting 2nd grade polynomial for phonon mode 13 at the $CrCl_2(pyz)_2$ system.

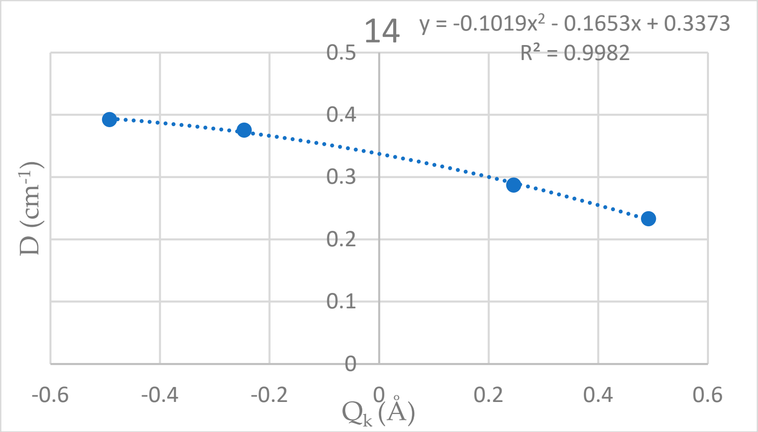

Figure S40: Calculated D values as a function of the distorted geometries Q_k with the fitting 2nd grade polynomial for phonon mode 14 at the $CrCl_2(pyz)_2$ system.

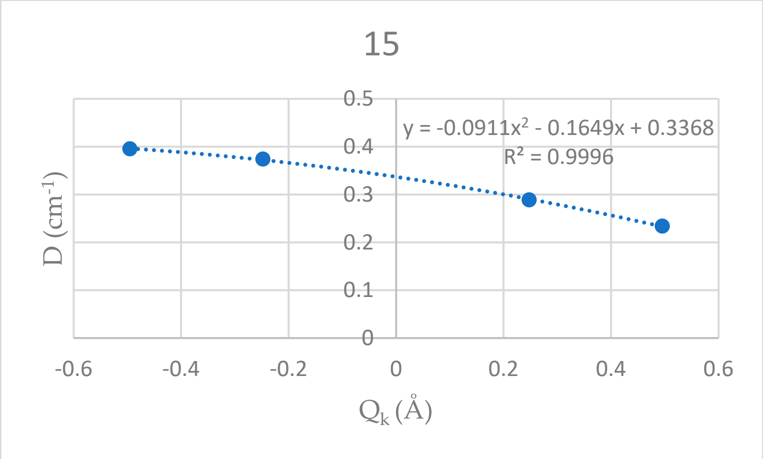

Figure S41: Calculated D values as a function of the distorted geometries Q_k with the fitting 2nd grade polynomial for phonon mode 15 at the $CrCl_2(pyz)_2$ system.

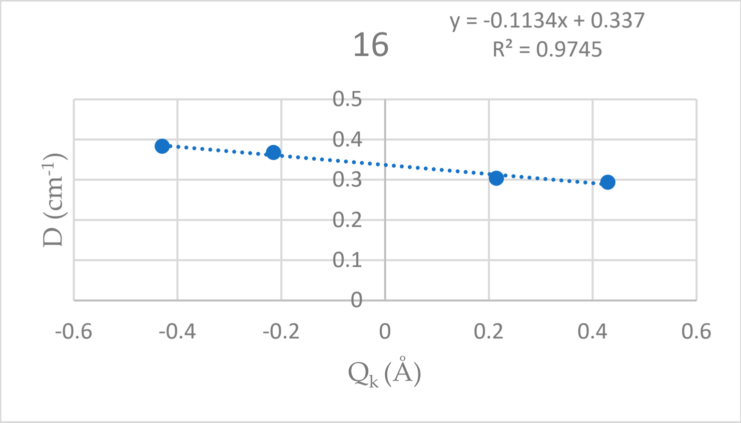

Figure S42: Calculated D values as a function of the distorted geometries Q_k with the fitting 2nd grade polynomial for phonon mode 16 at the $CrCl_2(pyz)_2$ system.

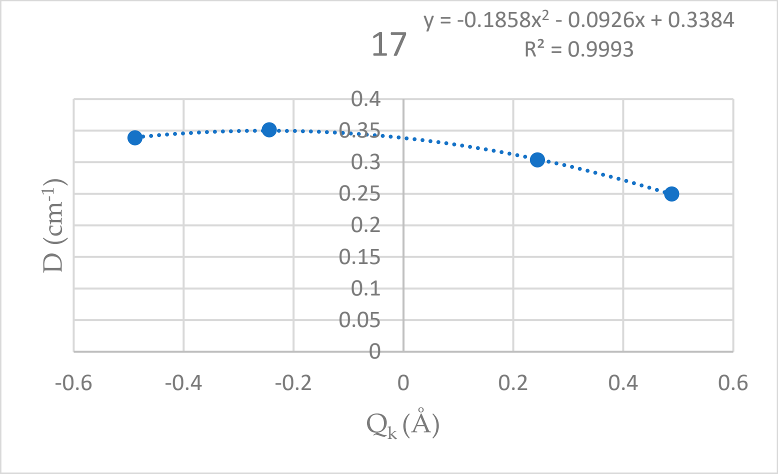

Figure S43: Calculated D values as a function of the distorted geometries Q_k with the fitting 2nd grade polynomial for phonon mode 17 at the $CrCl_2(pyz)_2$ system.

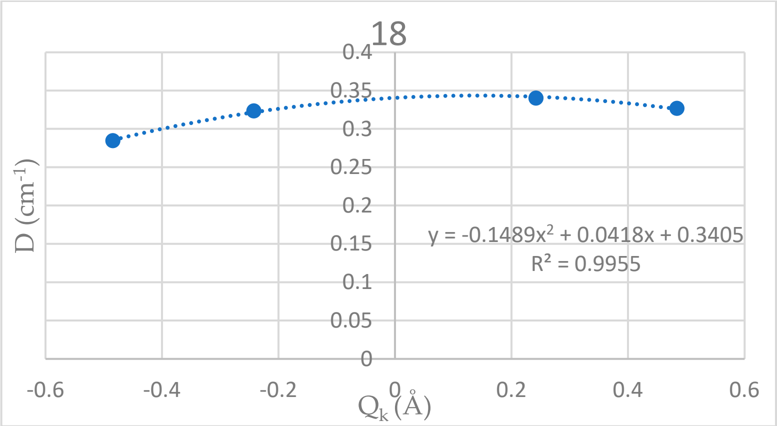

Figure S44: Calculated D values as a function of the distorted geometries Q_k with the fitting 2nd grade polynomial for phonon mode 18 at the $CrCl_2(pyz)_2$ system.

Table S5: $\left(\frac{\partial^2 D}{\partial Q_k^2}\right)_e$ calculated terms and Q_k for each phonon mode in $CrCl_2(pyz)_2$ system.

Phonon mode	$\left(\frac{\partial^2 D}{\partial Q_k^2}\right)_e$ (cm^{-1}/Å^2)	Q_k (Å)
1	-0.1794	±0.6870
2	-0.2208	±0.6286
3	-0.2484	±0.5403
4	-0.3452	±0.5322
5	-0.1932	±0.5312
6	-0.2572	±0.5056
7	-0.0308	±0.5091
8	-0.2334	±0.5002
9	-0.1892	±0.5033
10	-0.069	±0.4751
11	-0.123	±0.5843
12	-0.2198	±0.4959
13	-0.1614	±0.5246
14	-0.2038	±0.4918
15	-0.1822	±0.4952
16	-0.2268	±0.4293
17	-0.3716	±0.4882
18	-0.2978	±0.4842

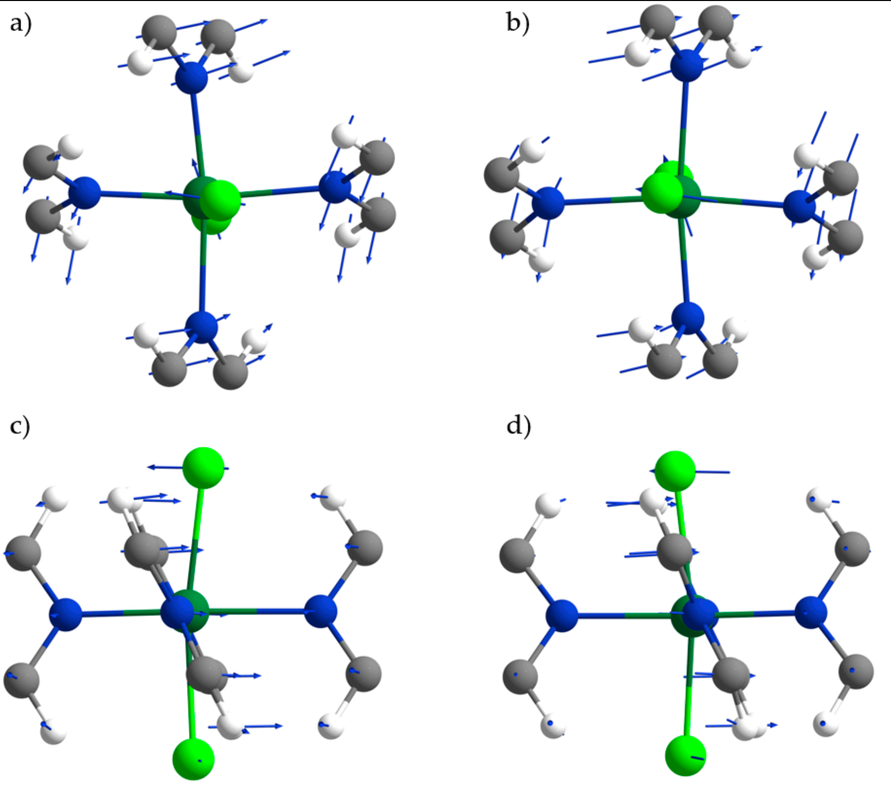

Figure S45: Graphical representation of phonon mode 6 on $CrCl_2(pyz)_2$ (a) top view of negative phase (b) top view of positive phase (c) side view of negative phase (d) side view of positive phase.

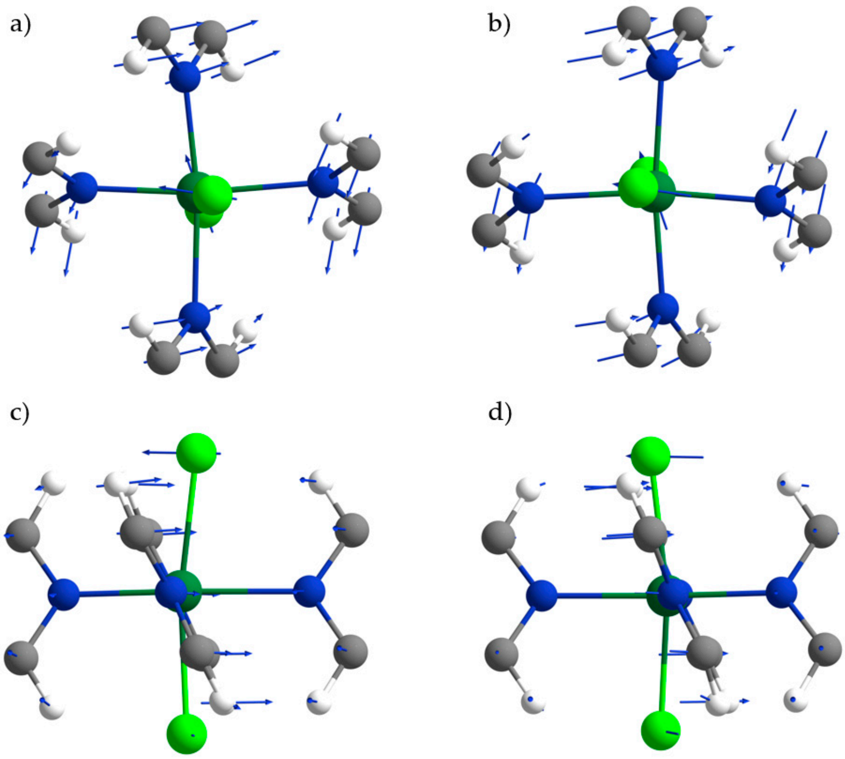

Figure S46: Graphical representation of phonon mode 10 on $CrCl_2(pyz)_2$ (a) top view of negative phase (b) top view of positive phase (c) side view of negative phase (d) side view of positive phase.

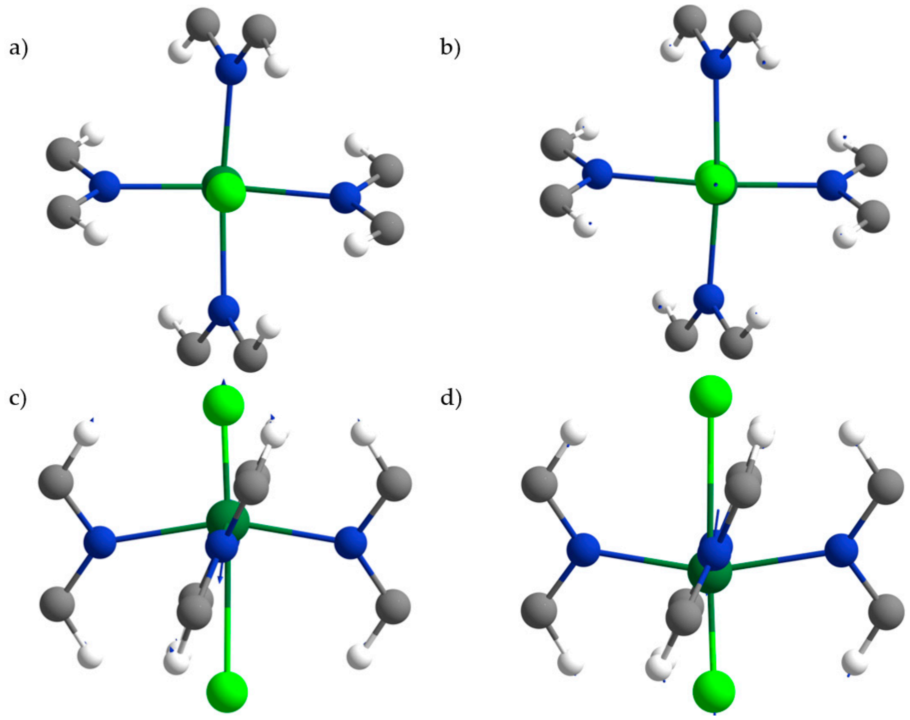

Figure S47: Graphical representation of phonon mode 18 on $CrCl_2(pyz)_2$ (a) top view of negative phase (b) top view of positive phase (c) side view of negative phase (d) side view of positive phase.
